# AARS2-catalyzed lactylation induces follicle development and premature ovarian insufficiency

**DOI:** 10.1038/s41420-025-02501-0

**Published:** 2025-04-29

**Authors:** Zhi-Ling Zhang, Shu-Ting Ren, Wan-Jie Yang, Xiao-Wen Xu, Shi-Min Zhao, Ke-Fei Fang, Yan Lin, Yi-Yuan Yuan, Xiao-Jin Zhang, Yun-Qin Chen, Wei Xu

**Affiliations:** 1 Obstetrics & Gynecology Hospital of Fudan University, State Key Laboratory of Genetic and Development of Complex Phenotypes, and Institutes of Biomedical Sciences, Fudan University, Shanghai, China; 2 Human Phenome Institute, Zhangjiang Fudan International Innovation Center, Fudan University, Shanghai, China; 3 Shanghai Institute of Cardiovascular Diseases, Zhongshan Hospital, Fudan University, Shanghai, China; 4 Shanghai Fifth People’s Hospital of Fudan University, Shanghai, China

**Keywords:** Post-translational modifications, Infertility

## Abstract

Lactate, a metabolite which is elevated in various developmental and pathological processes, exerts its signal through alanyl tRNA synthetases (AARS)-catalyzed protein lactylation. Herein, we report that elevated lactate and gain-of-function mitochondrial AARS (*AARS2*) mutations-induced hyper-lactylation promotes premature ovarian insufficiency (POI). Serum lactate is elevated in POI patients. POI-driving AARS2 mutations gain lactyltransferase activity. AARS2 lactylates and inactivates carnitine palmitoyl transferase 2 (CPT2), resulting in FFA accumulation that activates peroxisome proliferator-activated receptor γ (PPARγ), and potentiates follicle-stimulating hormone (FSH) to initiate follicle development. These, in synergy with the anabolites accumulation effects of AARS2, promoted lactylation-induced PDHA1 inactivation promote granular cell (GC) proliferation and primordial follicle development. GC-specific AARS2 overexpression does not affect primordial follicle number but speed up follicle depletion. AARS2 ablation or lactylation-inhibiting β-alanine treatments can prevent folliculogenesis and POI traits in mouse. These findings reveal that lactate signal drives follicle development, and inhibiting lactate signal could treat/prevent POI.

## Introduction

Primary ovarian insufficiency (POI) affects ~1% of women below 40 years of age, thereby severely impacting women’s fertility. Additionally, it significantly affects women’s mental health, as well as increases the risk of conditions such as osteoporosis, cardiovascular disease, and accelerated neurodegenerative aging [1, 2]. POI is associated with several factors, including genetic, environmental, immunological, and infectious [3]. POI-causing genes, such as *BMP15* [4], *GDF9* [5], *FOXL2* [6], and *P27* [7], encode proteins that play crucial roles in oocytes and granulosa cells (GCs) during folliculogenesis. Mutations in *FSHR* [8] and *FSHB* [9], which influence hormone signaling transduction in ovarian follicles, or *PTEN* [10] and *FOXO3a* [11], which maintain primordial follicles at resting state, can also cause POI.

Primordial follicle activation is initiated by GC proliferation followed by oocyte growth [12, 13]. GCs divide for at least 10 times in the growing follicles [14]. Additionally, follicle-stimulating hormone (FSH) functions at different developmental stages to ensure a staggered supply of maturing follicles across a woman’s reproductive lifespan [15]. FSH stimulates GC proliferation, particularly in antral follicles, by coordinating cell cycle regulators such as cyclin D2 and P27 [16, 17]. Responsiveness of GCs to FSH signaling can be augmented by FSHR overexpression [18].

Metabolic disorders, such as diabetes mellitus [19], are closely linked to POI onset, and inhibiting glycolysis using 2-deoxyglucose (2-DG) can prevent follicle activation [2]. Moreover, plasma lipid levels are often elevated in patients with POI [20], and obese women have shown a higher tendency to develop irregular menstrual cycles [21]. The ovarian reserve marker anti-Müllerian hormone (AMH) is decreased in obese women [22], and increased primordial follicle depletion has been observed in the ovaries of obese mice [23]. These findings collectively suggest that metabolism disorders, including dysregulated glucose and fatty acid metabolism, may be a causal factor in POI.

Lactate, the end product of glycolysis, is critical for development, include follicular development. Approximately 90% of consumed glucose produces lactate in implanting blastocysts (around 16 mM in mouse and 100 mM in human) [24, 25]. Studies have shown that lactate enhances mouse embryonic stem (ES) cell differentiation in vitro [26] and promotes sensory neural development in the otic vesicle [27]. High levels of lactate production can promote radial glial progenitor proliferation and vessel growth in developing embryonic neocortex [28]. The concentration of follicular fluid lactate is ~6.27 mM in humans [29] and 17.3 mM in mice [30], which can be increased by FSH or luteinizing hormone (LH) stimulation [31]. Mouse ovarian follicles produce large amounts of lactate to support their growth and maturation in vitro [31, 32], whereas reduced follicular fluid lactate is associated with poor developmental potential of oocytes [33].

Lactate modifies proteins to regulate various physiological and pathological processes [34–37], including cancer onset and organ development. We previously identified mitochondrial alanyl-tRNA synthetase 2 (*AARS2*) as a bona fide protein lactyltransferase [38]. Remarkably, genome-wide array data have indicated that *AARS2* is a core gene determining ovarian aging [39]. Moreover, mutations in *AARS2* have been found to be associated with POI [40–51], suggesting that AARS2-generated lactate affects follicle development. However, the precise molecular mechanisms by which lactate signaling, mediated by AARS2-catalyzed protein lactylation, specifically affects follicle development and contributes to POI remain unclear. In the present study, we analyzed the correlation of increased lactate signal in human POI samples and confirmed that *AARS2* mutants with lactyltransferase activity promote POI.

## Results

### Lactate and fatty acids are elevated in POI patients

**Fig. 1:**
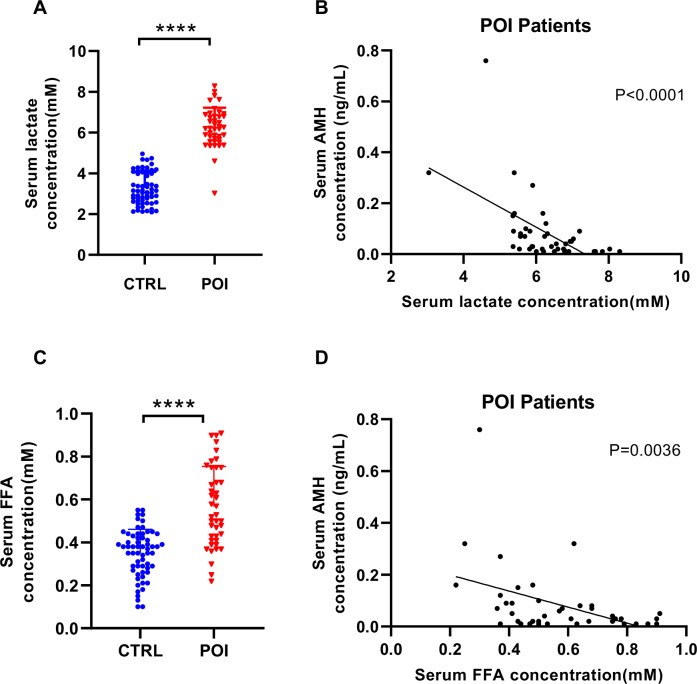
Lactate and fatty acid levels are elevated in POI patients. Serum lactate and fatty acids levels were measured in 42 POI patients and 61 age-matched healthy individuals (**A**, **C**), and lactate and fatty acids levels were plotted against the functional ovarian reserve indicator AMH (**B**, **D**). The following symbols have been used for indicating statistical analysis throughout the Figures: *P < 0.05, **P < 0.005, ***P < 0.001, ****P < 0.0001, NS not significant.

To confirm that metabolic signals are involved in POI onset, we compared sera metabolomes between 42 POI patients and 61 age-matched healthy individuals (Table S1). Serum lactate was positively correlated to POI (Fig. 1A) and negatively correlated to follicular reserve AMH (Fig. 1B), which reflects the pool of growing follicles that potentially can ovulate, suggesting that lactate contributes to early follicle development. Serum free fatty acids levels were also positively correlated to POI (Fig. 1C) and negatively correlated to follicular reserve AMH (Fig. 1D). These reaffirmed that lactate and fatty acids deregulation is correlated to POI onset.

### GC-specific AARS2 overexpression induces POI in mice

We investigated whether AARS2, which generates lactate signals by catalyzing lysine lactylation (LacK) [38] and is frequently mutated in POI [40–51], is involved in POI onset. Human lactyltransferase AARS2 (hAARS2) was specifically expressed in mouse GCs (*AARS2* GOE) to mimic an enhanced lactate signal (Fig. S1A), and *Aars2* was knocked out from mouse GCs (*Aars2* GKO) to mimic a decreased lactate signal (Fig. S1B).

**Fig. 2:**
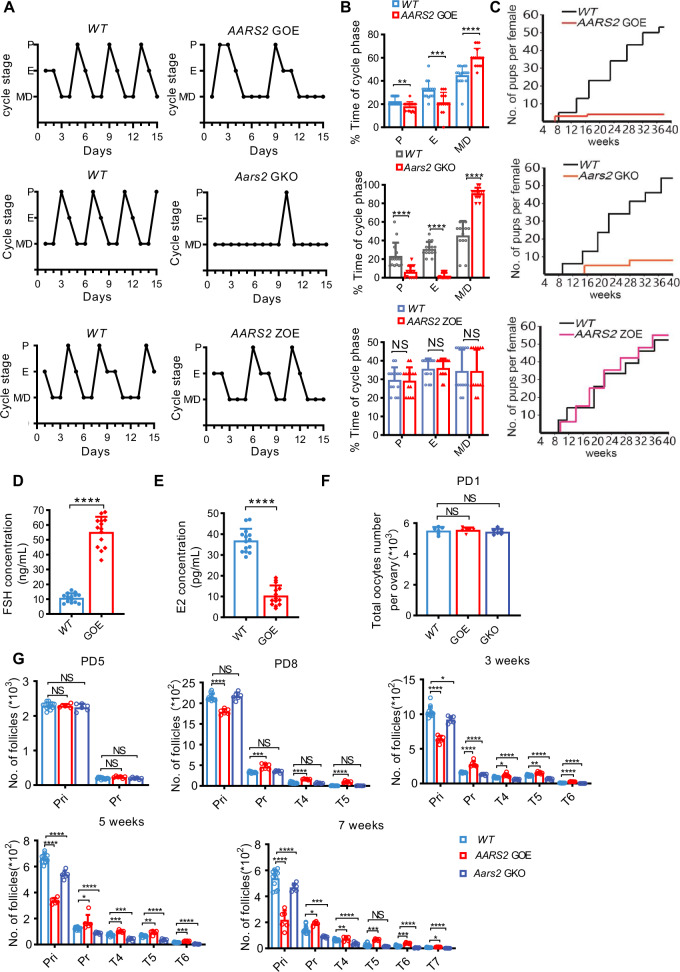
GC-specific AARS2 overexpression induces mouse POI. **A**–**B** AARS2 regulates estrous cyclicity. Representative estrous cyclicity of C57, *AARS2* GOE, *Aars2* GKO, and *AARS2* ZOE mice during 15 consecutive days since postnatal 8 weeks (**A**) and the cycle phase times (*n* = 16 for *AARS2* GOE and 14 for *Aars2* GKO and *AARS2* ZOE) represented as mean ± SEM (**B**). **C** AARS2 affects the litter size. The cumulative number of pups in C57, *AARS2* GOE, *Aars2* GKO, and *AARS2* ZOE was analyzed (*n* = 6). **D**–**E** Levels of FSH (**D**) and E2 (**E**) in adult *AARS2* GOE and *WT* mice (16–20 weeks old, *n* = 14) were measured. **F** AARS2 does not affect oocyte number. The number of oocytes in C57*, AARS2* GOE, and *Aars2* GKO mice (*n* = 6, one ovary from each mouse) was measured at PD1. See also Fig. S2. **G** AARS2 affects ovarian follicle number. The ovarian follicles in *AARS2* GOE*, Aars2* GKO, and C57 mice were quantified (*n* = 6) at PD5, 8, and PD3-, 5-, and 7-weeks. Numbers of primordial (Pri), primary (Pr), type 4 (T4), type 5 (T5), type 6 (T6), and type 7 (T7) follicles were counted (mean ± SEM). See also Fig. S2.

*AARS2* GOE and *Aars2* GKO mice had comparable weight (Fig. S1C), food consumption (Fig. S1D), and blood glucose levels (Fig. S1E) to wild-type C57 mice. However, *AARS2* GOE mice exhibited altered estrous cyclicity after sex maturation at week 8 (Fig. 2A), displaying extended metestrus and diestrus and shortened estrus (Fig. 2B). Moreover, these mice had decreased reproductive ability, evidenced by reduced litter size compared with wildtype females (Fig. 2C). These findings, along with the results that oocyte-specific AARS2-overexpressing mouse (Fig. S1F, *AARS2* ZOE mouse) had regular estrous cyclicity (Fig. 2A) and normal litter size (Fig. 2C), suggest that AARS2 mainly acts on GCs to regulate mouse reproductivity. In contrast, *Aars2* GKO mice had diminished estrous cyclicity (Fig. 2A) and an average litter size of 6 (Fig. 2C). These results suggest that high AARS2, i.e., enhanced lactate signal, in GCs decreases mouse reproductivity.

POI signatures [52], namely upregulation of FSH and downregulation of estradiol E2, were found in *AARS2* GOE mice (Fig. 2D, E), suggesting that the decreased reproductivity of *AARS2* GOE mice might have been due to POI development. The number of oocytes on postnatal day 1 (PD1) and primordial (Pri) and primary (Pr) follicles on PD5 in C57, *AARS2* GOE, and *Aars2* GKO mice ovaries were comparable (Figs. 2F, G, and S2A, B), suggesting that GC AARS2 levels had no impact on primordial follicle generation and follicle development initially. However, when mice were older, more developed follicles and fewer primordial follicles, which started disappearing from PD16-weeks onwards, were found in *AARS2* GOE mouse ovaries than in C57 mouse ovaries (Figs. 2G and S2B), suggesting that lactate signal drives follicle development and depletes primordial follicles. This was substantiated by the observation that developed and primordial follicles at PD7 weeks were lower in *Aars2* GKO mouse ovaries than in C57 mouse ovaries (Figs. 2G and S2B). Moreover, an increase in zona pellucida remnants, typically remnants of oocytes that have undergone atresia after synthesis of the zona pellucida, was observed in *Aars2* GKO mice at PD7 weeks (Fig. S2B). These results collectively indicate that enhanced lactate signal in GCs promotes the development and depletion of follicles.

### POI-causing AARS2 mutants gain lactyltransferase activities

**Fig. 3:**
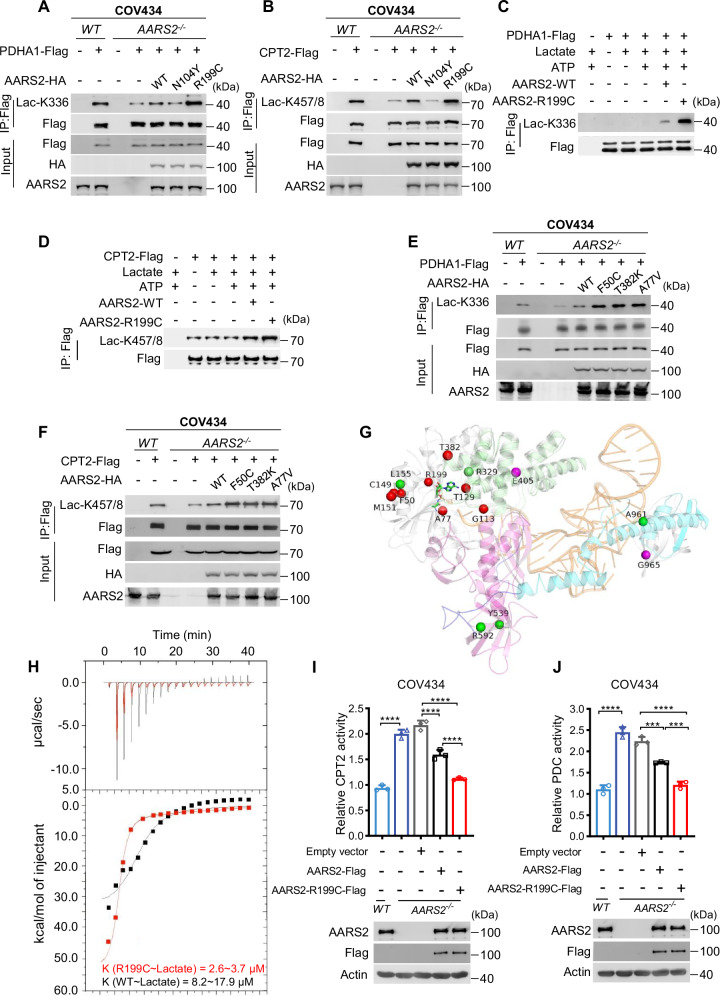
POI-causing AARS2 mutants gain lactyltransferase activities. **A**–**B** R199C gained lactyltransferase activity. Lac-K336 levels (**A**) and Lac-K457/8 levels (**B**) of PDHA1-Flag and CPT2-Flag that were expressed from *AARS2*^*−/−*^COV434, and from *AARS2*^*−/−*^ COV434 that co-expressed AARS2, N104Y, or R199C were detected. **C**–**D** R199C has stronger lactyltransferase activity than AARS2. The abilities to form Lac-K336 in purified PDHA1 (**C**) and Lac-K457/8 in purified CPT2 (**D**) of AARS2 and R199C were detected in vitro. **E**–**F** All POI-causing AARS2 mutants gained lactyltransferase activity. Lac-K336 (**E**) and Lac-K457/8 (**F**) levels were detected in immunoprecipitated PDHA1-Flag and CPT2-Flag purified from COV434, *AARS2*^*−/−*^ COV434, and *AARS2*^*−/−*^ COV434 cells ectopically expressing AARS2, F50C, T328K, or A77V. **G** POI-causing AARS2 mutations cluster around substrate-binding pocket. Disease-causing sites are marked in human AARS2 structure. POI-associated mutations are shown as spheres and labeled in red, cardiomyopathy-associated mutations are labeled in green, and leukodystrophy-associated mutations are labeled in violet. Aminoacylation subdomain (35–312 aa, gray) is in gray, tRNA recognition subdomain (313–477 aa) is in green, linker (478–529 aa) between tRNA recognition and editing domain is in blue, and editing domain (530–783 aa) and C-terminal domain (784-985) are in magenta and cyan, respectively. Backbone of docked tRNA and alanyl-adenylate are shown in orange and rainbow, respectively. **H** R199C has stronger binding ability for lactate. Isothermal Titration Calorimetry (ITC) was employed to measure the binding affinity of lactate to AARS2 (balk line) and R199C (red line). **I**–**J** R199C inhibits CPT2 and PDHA1 more strongly than AARS2. CPT2 (**I**) and PDC (**J**) specific activities were detected in purified CPT2 and cell lysates from COV434, *AARS2*^*−/−*^ COV434, and *AARS2*^*−/−*^ COV434 that co-expressed AARS2 or R199C, respectively (*n* = 3).

We analyzed the lactate signal-generating ability, i.e., lactyltransferase activities of POI-related AARS2 mutations, including the most frequently reported mutation AARS2^R199C^ (R199C) [53], which has no impact in alanyl tRNA charging activities [46]. AARS2 deletion abrogated PDHA1 K336 and CPT2 K457/8 lactylation in the human granulosa cell line COV343 [38], and R199C exhibited a higher ability to lactylate PDHA1 K336 and CPT2 K457/8 in *AARS2*^*−/−*^ COV343 cells (Fig. 3A, B). We also found that R199C exhibited an increased ability to lactylate synthetic K336-containing PDHA1 and K457/8-containing CPT2 peptides (Fig. S3A, B) and intact recombinant PDHA1 and CPT2 proteins in vitro (Fig. 3C, D). Moreover, other POI-related mutations, including F50C, T382K, and A77V, had higher ability to lactylate PDHA1 K336 and CPT2 K457/8 than AARS2 in *AARS2*^*−/−*^ COV343 cells (Fig. 3E, F). These results suggest that increased lactyltransferase activities associated with AARS2 mutations primarily drive their POI-promoting effects.

That POI-driving AARS2 mutations share similar mechanism to promote POI is supported by the fact that their mutated sites were all adjacent to AARS2 substrates-binding pocket (Fig. 3G), potentially facilitating their favorable binding to lactate. This hypothesis was further confirmed by an Isothermal Titration Calorimetry (ITC) analysis, which revealed that R199C has a stronger ability to bind lactate than AARS2 (Fig. 3H), while R199C and AARS2 had comparable ability to interact with PDHA1 and CPT2 (Fig. S3C, D). Further support for the gain of lactyltransferase activity in R199C was demonstrated by overexpressing R199C, which inhibited CPT2 (Fig. 3I) and PDHA1 (Fig. 3J) specific activities more potently when they were ectopically expressed in *AARS2*^*−/−*^ COV343 cells. Additionally, both R199C and AARS2 exhibited comparable capacities for estrogen biosynthesis in *AARS2*^*−/−*^ COV434 cells (Fig. S3E). Based on these observations, we propose that the decreased E2 level in GOE mice may be attributed to a deficiency in functional follicles. This deficiency is likely a consequence of lactylation-driven accelerated folliculogenesis, which ultimately leads to premature follicle depletion.

### R199C is a stronger follicle development inducer than AARS2

**Fig. 4:**
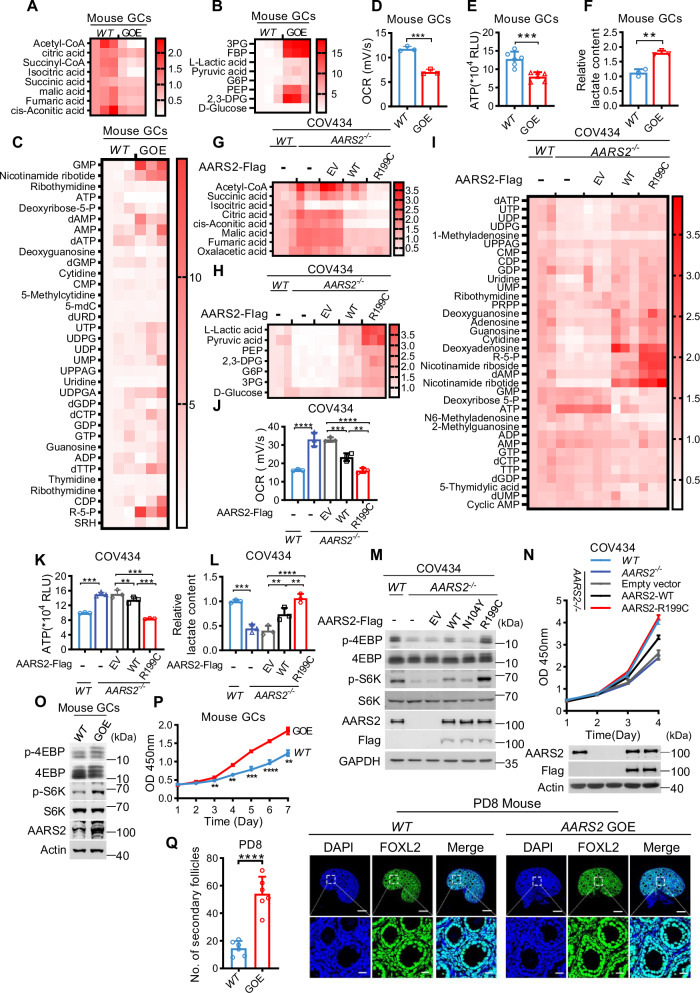
R199C is a stronger follicle development inducer than AARS2. **A**–**F** AARS2 inhibits the TCA cycle and accumulates anabolites in GCs. Heatmap of annotated metabolites in TCA cycle (**A**), glycolysis (**B**), and PPP (**C**). The inhibition of the TCA cycle is further confirmed by measuring OCR (**D**), ATP (**E**), and lactate production (**F**) in GCs of C57 and GOE mice. **G–L** R199C has a strong ability to reprogram GC metabolism. The metabolites changes in TCA cycle (**G**), glycolysis (**H**), PPP (**I**), and OCR (**J**), ATP (**K**), and lactate (**L**) were compared between *AARS2*^*−/−*^ COV434 cells and *AARS2*^*−/−*^ COV434 cells expressing either AARS2 or R199C. **M** AARS2 activates mTORC1 signaling in COV434 cells. p-S6K and p-4EBP were detected in COV434 cells, *AARS2*^*−/−*^ COV434 cells, *AARS2*^*−/−*^ COV434 cells expressing AARS2, and AARS2 mutants. **N** AARS2 promotes cell proliferation in COV434 cells. CCK-8 assay was employed to measure growth of COV434, *AARS2*^*−/−*^ COV434 cells, and *AARS2*^*−/−*^ COV434 expressing AARS2 or R199C. **O** AARS2 activates mTORC1 signaling in mouse GCs. p-S6K and p-4EBP were detected in C57 and *AARS2* GOE mouse GCs. **P** AARS2 promotes cell proliferation in mouse GCs. CCK-8 assay was employed to measure growth of GCs from C57 and *AARS2* GOE mice. **Q** AARS2 promotes mouse follicular development. Representative images of PD8-C57 and *AARS2* GOE mice ovarian sections, stained for DAPI, FOXL2 (right panel). The secondary follicles in 6 mice ovaries were counted (left panel) (scale bars, 200 μm (top panel), 20 μm (bottom panel)).

*AARS2* GOE mouse GCs had lower the tricarboxylic acid (TCA) intermediate metabolites but higher glycolytic/pentose phosphate pathway (PPP) intermediate metabolites, i.e., anabolites, than wildtype mice GCs (Fig. 4A–C). Additionally, *AARS2* GOE mouse GCs had decreased oxygen consumption rate (OCR) (Fig. 4D), lower ATP production (Fig. 4E), and elevated lactate levels (Fig. 4F). These findings align with the concept that PDHA1 lactylation inhibits OXPHOS and reserves anabolites for anabolism [38] and suggest that AARS2 may promote GC anabolism. Intriguingly, these metabolic reprograming effects were more markedly induced by R199C overexpression than AARS2 overexpression in COV434 cells (Fig. 4G–L), suggesting that R199C may be a stronger GC anabolism inducer than AARS2.

The initiation of follicle development/maturation begins after mTORC1 signaling activation, which promotes GC proliferation [13] and follicle development [54]. We compared mTORC1 signaling activation and growth potency of AARS2 and R199C in *AARS2*^*−/−*^ COV434 cells. When expressed at comparable levels, R199C was a more potent activator of mTORC1 than AARS2 in *AARS2*^*−/−*^ COV434 cells (Fig. 4M), consistent with the fact that high anabolites activate mTORC1 [55, 56]. Moreover, R199C restored *AARS2*^*−/−*^ COV434 cell growth more effectively than AARS2 when overexpressed at similar levels (Fig. 4N). Furthermore, primary GCs of *AARS2* GOE mice exhibited enhanced mTORC1 signaling (Fig. 4O) and faster proliferation than those of *WT* mice (Fig. 4P), consistent with the observation that the granulosa marker FOXL2 is more strongly expressed in the ovaries of *AARS2* GOE female PD8 mice compared with *WT* mice (Fig. 4Q). These results were in agreement with the notion that mTORC1 activation stimulates GC proliferation and follicle development. Notably, silencing PDHA1, but not CPT2, activated the mTORC1 signaling in COV434 cells (Fig. S4A) and blunted AARS2 overexpression to activate the mTORC1 signaling in *AARS2*^*−/−*^ COV434 cells (Fig. S4B). This aligns with the fact that accumulation of glycolytic intermediates activates mTORC1 signaling [56] and that lactate signal activates mTORC1 signaling mainly by inactivating PDHA1. In conclusion, overactivated mTORC1 by AARS2 or R199C overexpression facilitates anabolite accumulation that activates follicle development via mTORC1 activation-induced GC proliferation.

### Lactate signal activates PPARγ to potentiate FSH action

In line with lactylation’s role in inactivating CPT2 and FAO [38], free fatty acid (FFA) level was increased and decreased in the ovaries of *AARS2* GOE and *Aars2* GKO mice, respectively (Fig. S5A, B). The accumulation of FFA was further supported by increased and decreased lipid droplet in ovaries/GCs of *AARS2* GOE and *Aars2* GKO mice, respectively (Fig. S5C–F).

**Fig. 5:**
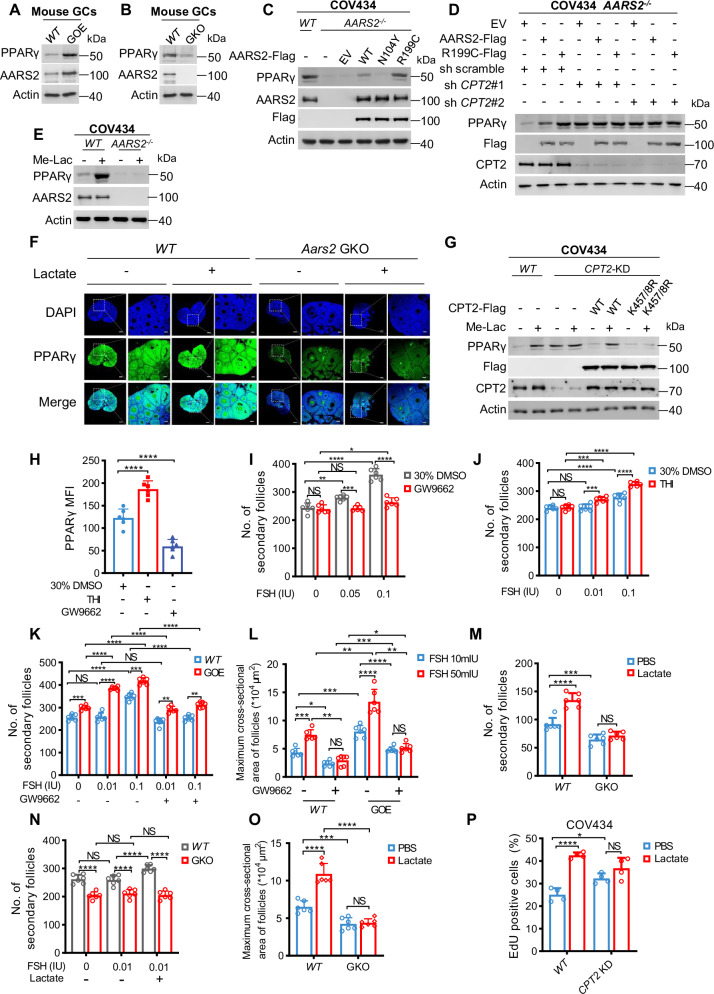
Lactate signal activates PPARγ to potentiate FSH action. **A–B** AARS2 induces PPARγ in mouse GCs. The levels of PPARγ were compared between C57 and *AARS2* GOE (**A**) and between C57 and *Aars2* GKO (**B**) mouse GCs. **C** PPARγ was detected in COV434 cells, *AARS2*^*−/−*^ COV434 cells and in *AARS2*^*−/−*^ COV434 cells expressing either AARS2 or AARS2 mutations. **D** AARS2 CPT2-dependently induces PPARγ. The abilities of AARS2 and R199C to induce PPARγ were detected in *AARS2*^*−/−*^ COV434 cells and *AARS2*^*−/−*^ COV434 cells with silenced *CPT2* through shRNAs. **E** Lactate induces PPARγ in cells. The PPARγ levels were detected in COV434 cells and *AARS2*^*−/−*^ COV434 cells with or without methyl L-lactate treatments. **F** Lactate induces PPARγ in mouse ovaries. The PPARγ levels were stained in ovarian sections of C57 and *Aars2* GKO mice that were intraperitoneally injected with either PBS or 20 mg/kg lactate (scale bars, 200 μm (left panel), 50 μm (right panel)). **G** CPT2 lactylation accounts for lactate’s effects on inducing PPARγ. The PPARγ levels were detected in COV434 cells and *CPT2* KD COV434 cells that were transfected with CPT2 and lactylation null CPT2^K457/8R^. **H** THI increases and GW9662 decreases PPARγ signaling in mouse ovaries. Mean fluorescence intensity (MFI) of PPARγ signaling in ovarian sections of mice intraperitoneal injected with 30% DMSO, THI, or GW9662 (*n* = 6) was analyzed and represented as mean ± SEM. See also Fig. S6. **I**, **J** PPARγ inhibition diminished, and PPARγ activated FSH to promote mouse follicle development. Ovarian secondary follicles were counted in *WT* mice intraperitoneal injected with indicated FSH together with either 30% DMSO or GW9662 dissolved in 30% DMSO (**I**), and with either 30% DMSO or THI dissolved in 30% DMSO (**J**). Data are represented as mean ± SEM, *n* = 6. See also Fig. S6. **K** AARS2 overexpression promotes mouse follicle development. Ovarian secondary follicles were counted in *WT* and *AARS2* GOE mice that were intraperitoneal injected with indicated FSH together with either 30% DMSO and GW9662 dissolved in 30% DMSO. Data are represented as mean ± SEM, *n* = 6. See also Fig. S6. **L** AARS2 promotes follicle growth in vitro. The maximum cross-sectional area of in vitro cultured *WT* and *AARS2* GOE mouse follicles, with presence of gradient FSH, and under absence or presence of GW9662 was quantified. Data are represented as mean ± SEM, *n* = 6. See also Fig. S6. **M** Lactate promotes follicle development in mouse ovaries in an AARS2-dependent manner. Ovarian secondary follicles of *WT* and *Aars2* GKO mice with or without intraperitoneal lactate injection were counted. Data are represented as mean ± SEM, *n* = 6. See also Fig. S6. **N** Lactate potentiates FSH to initiate follicle development in an AARS2-dependent manner. Ovarian secondary follicles of *WT* and *Aars2* GKO mice that were with or without FSH, or lactate together with FSH, were quantified, *n* = 6. See also Fig. S6. **O** Lactate AARS2-dependently promotes follicles growth in vitro. The maximum cross-sectional area of *WT* and *AARS2* GOE mice follicles cultured under with or without lactate in the culture media was quantified, *n* = 6. See also Fig. S6. **P** Lactate CPT2-dependently increases EdU incorporation. EdU positive cells in *WT* and *CPT2* KD COV434 cells with or without lactate treatment were analyzed, *n* = 6. See also Fig. S6.

Fatty acids-activated PPARγ, the major PPAR expressed in GCs that has known follicle development-promoting ability [57], was activated and inactivated in the ovaries of *AARS2* GOE and *Aars2* GKO mice, respectively (Fig. 5A, B). The inactivated PPARγ in *AARS2*^*−/−*^ COV434 cells could be rescued by AARS2 and R199C overexpression, but not by catalytic dead AARS2^N104Y^ overexpression; moreover, when expressed at similar levels, R199C showed higher efficacy in reactivating PPARγ than AARS2 in *AARS2*^*−/−*^ COV434 cells (Fig. 5C), confirming that lactate signal activates PPARγ signaling. However, the ability of AARS2 and R199C expression to activate PPARγ in *AARS2*^*−/−*^ COV434 cells was abrogated by CPT2 silencing (Fig. 5D), consistent with our findings that AARS2 activates PPARγ signaling by inactivating CPT2.

Lactate increased PPARγ expression level in wildtype but not *AARS2*^*−/−*^ COV434 cells (Fig. 5E). Intraperitoneal lactate injection increased PPARγ signaling in ovaries of *WT* mice but not in ovaries of *Aars2* GKO mice (Fig. 5F). Moreover, *CPT2-*KD in COV434 cells caused PPARγ upregulation and resistance to lactate treatment, and this resistance was relieved by CPT2 but not lactylation-null CPT2^K457/8R^ mutant putback (Fig. 5G). Taken together, these results support that lactate signal activates PPARγ signaling by inactivating CPT2.

Intraperitoneal injection of the PPARγ inhibitor GW9662 in C57 mouse inhibited PPARγ signaling (Figs. 5H and S6A) and retarded the responses of follicle development to high concentrations of FSH (Figs. 5I and S6B). Moreover, the PPARγ activator thiazolidinediones (THI) [58] potentiated FSH to increase FOXL2 signaling in ovaries of *WT* mice (Figs. 5H, J and S6C). These results confirmed that PPARγ signaling interacts with FSH signaling to activate follicle development [57].

FSH activated FOXL2 signaling at much lower doses in ovaries of *AARS2* GOE mice compared with C57 wildtype mice, and GW9662 inhibited FOXL2 signaling in ovaries of both mice (Figs. 5K and S6D). Moreover, FOXL2 signal in in vitro-cultured primary follicles from wildtype C57 mouse was lower compared with those from *AARS2* GOE mouse, when GW9662 was used as an inhibitor, both genotypes displayed decreased FOXL2 signaling (Figs. 5L and S6E). These results suggest that AARS2 potentiates follicle development.

FOXL2 signaling in ovaries of C57 mouse was activated by 20 mg/kg intraperitoneal lactate injection (Figs. 5M and S6F), and lactate injection potentiated FSH to induce follicle development in C57 but not in *Aars2*^*−/−*^ mice (Figs. 5N and S6G). Similarly, responses of FOXL2 signal in in vitro-cultured primary follicles from wildtype C57 mouse to FSH were sensitized by lactate treatment, and this effect was not observed in cultured follicles of *Aars2*^*−/−*^ mice (Figs. 5O and S6H). Moreover, lactate failed to increase EdU incorporation, a proliferation marker, in *CPT2* KD COV434 cells (Figs. 5P and S6I). These findings collectively validate that lactate signal sensitizes FSH actions through CPT2 inactivation and consequently PPARγ activation.

### Hyper-lactylation induces depletion of premature follicles

**Fig. 6:**
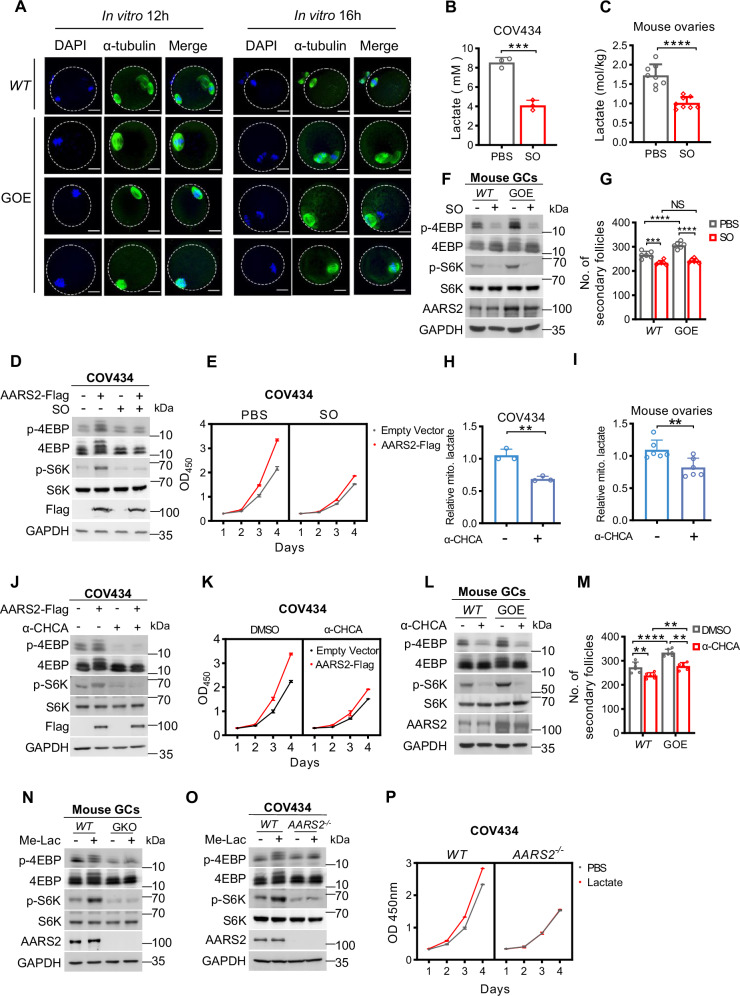
High lactate signal promotes follicles depletion. **A** AARS2 overexpression induces meiotic maturation defects. Spindle morphologies and chromosome alignment of in vitro C57 and *AARS2* GOE mouse oocytes were monitored at 12 and 16 h after culturing. DNA was stained with DAPI, and spindle was stained with α-tubulin (scale bars, 20 μm). **B**–**C** SO treatment decreases lactate levels. Lactate concentration in COV434 cells (**B**, *n* = 3) and in mouse ovary (**C**, *n* = 6) were detected with or without the LDH inhibitor SO treatment. **D**–**F** Lactylation activates mTORC1 and GC proliferation. The effects of SO on p-S6K and p-4EBP (**D**) and growth (**E**, *n* = 3) were detected in COV434 cells and AARS2-overexpressing COV434 cells, and in C57 and *AARS2* GOE mice GCs (**F**). **G** Lactylation regulates follicle development. The FOXL2 levels in ovaries of C57 and *AARS2* GOE mice intraperitoneally injected with PBS or SO were quantified (right, *n* = 6). **H**–**I** *α*-CHCA treatment decreased mitochondrial lactate. Mitochondrial lactate was detected in COV434 cells (**H**) and Mouse ovaries (**I**) with or without *α*-CHCA treatment. **J**–**L** Lactylation activates mTORC1 and GC proliferation. The effects of α-CHCA on p-S6K and p-4EBP (**J**) and growth (**K**, *n* = 3) were detected in COV434 cells and AARS2-overexpressing COV434 cells, and in C57 and *AARS2* GOE mice GCs (**L**). **M** Lactylation regulates follicle development. The FOXL2 levels in ovaries of C57 and *AARS2* GOE mice intraperitoneally injected with DMSO or α-CHCA were quantified (right, *n* = 6). **N**–**O** Lactate AARS2-dependently activates GC mTORC1. p-S6K and p-4EBP were detected in GCs of C57 and *Aars2* GKO mice (**N**) and in COV434 cells and *AARS2*^*−/−*^ COV434 cells (**O**), which were cultured with or without methyl L-lactate. **P** Lactate AARS2-dependently promotes GC proliferation. Proliferation of COV434 cells and *AARS2*^*−*/−^ COV434 cells was measured with or without methyl L-lactate in the culture media.

At 3 weeks of age, more secondary follicles, quantified with 2 or more layers of GCs in follicles (see Fig. 2G) and higher percentage of abnormal oocytes (Fig. 6A), were observed in *AARS2* GOE mice. LDH inhibitor sodium oxamate (SO) treatment, which decreased lactate levels in COV434 cells and in ovaries of mice as well as decreased PDHA1 and CPT2 lactylation (Figs. 6B, C, and S7A, B), inhibited both mTORC1 signaling and proliferation in AARS2-overexpressing COV434 cells (Fig. 6D, E) and suppressed mTORC1 and FOXL2 signaling in GCs/ovaries of *AARS2* GOE mice (Figs. 6F, G, and S7C). In addition, treatment with MCT1 inhibitor α-CHCA (α-cyano-4hydroxycinnamic acid) effectively decreased mitochondrial lactate levels in COV434 cells and in mouse ovaries (Fig. 6H–I) as previously reported [38]. This treatment also reduced lactylation of PDHA1 and CPT2 in COV434 cells, as well as the overal mitochondrial lactylation level in mouse ovaries (Fig. S7D–F). Importantly, α-CHCA treatment inhibited both mTORC1 signaling and cell proliferation in AARS2-overexpressing COV434 cells (Fig. 6J-K). furthermore, it suppressed mTORC1 and FOXL2 signaling in GCs and ovaries of *AARS2* GOE mice (Figs. 6L, M, and S7G). Finally, lactate activated mTORC1 signaling in C57 but not in *Aars2* GKO mouse GCs (Fig. 6N). Lactate activated mTORC1 signaling and stimulated proliferation in COV434, but not in *AARS2*^*−/−*^ COV434 cells (Fig. 6O, P). These results confirm that lactate signal in GCs induces premature follicle development and follicle depletion. Interestingly, GCs express MCT1 and MCT4, and their expression levels increase significantly during folliculogenesis (Fig. S7H–I). This upregulation may indicate that granulosa cells have a high demand for lactate uptake and secretion during follicle development.

### Inhibiting AARS2-catalyzed lactylation prevents POI traits

**Fig. 7:**
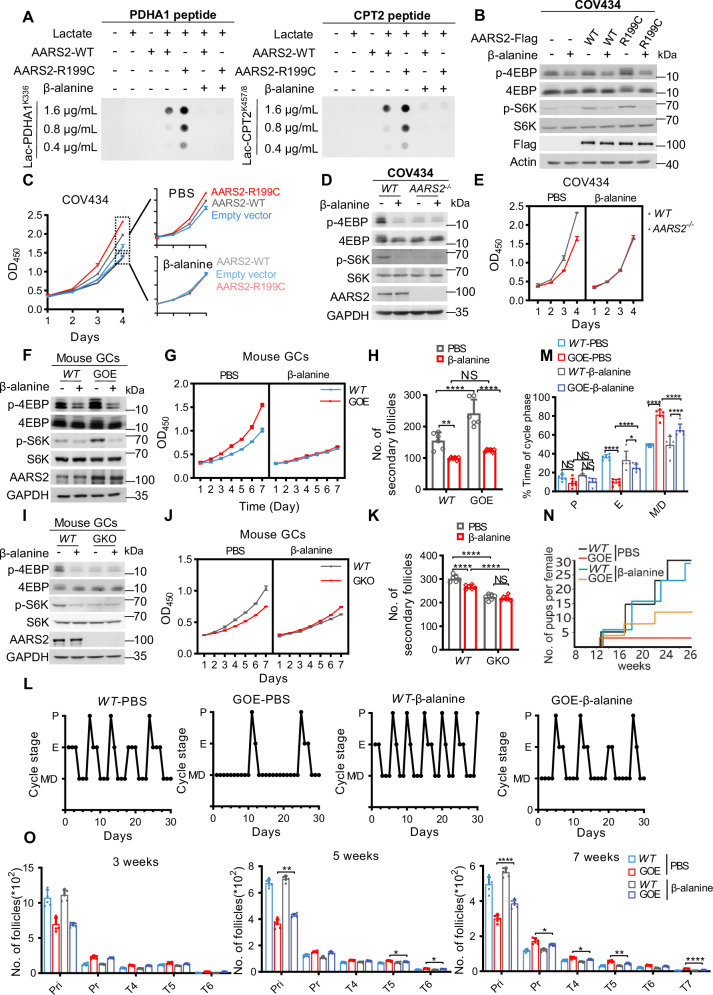
Downregulating lactate signal prevents POI traits in AARS2 GOE mice. **A** β-alanine inhibits AARS2- and R199C-catalyzed PDHA1 and CPT2 lactylation in vitro. In vitro AARS2- and R199C-catalyzed PDHA1 (left) and CPT2 (right) lactylation were detected when β-alanine was present and absent in the reaction mix. **B**–**C** β-alanine inhibits AARS2- and R199C-induced mTORC1 signaling. p-S6K and p-4EBP (**B**) and cell proliferation (**C**, *n* = 3) were detected in COV434 cells and AARS2-overexpressing COV434 cells that were cultured with or without β-alanine. **D**–**E** *AARS2* deletion abrogated β-alanine to inhibit GC mTORC1 and proliferation. The β-alanine effects on p-S6K and p-4EBP (**D**), and proliferation (**E**, *n* = 3) of COV434 cells and *AARS2*^*−/−*^ COV434 cells were detected. **F–H** β-alanine inhibits follicle development. The β-alanine effects on p-S6K and p-4EBP (**F**), GC proliferation (**G**, *n* = 3), and number of secondary follicles (**H**, *n* = 6) of C57 and *AARS2* GOE mice were detected. See also Fig. S7. **I–K** β-alanine AARS2*-*dependently inhibits follicle development. The effects of β-alanine on p-S6K and p-4EBP (**I**), GC proliferation (**J**, *n* = 3), and number of secondary follicles of C57 and *Aars2* GKO mice (**K**, *n* = 6) were detected. See also Fig. S7. **L–N** β-alanine relieves POI traits in *AARS2* GOE mice. The effects of intraperitoneally injected β-alanine on estrous cyclicity (**L**) and the cycle phase times (**M**) of C57 and *AARS2* GOE mice were detected, *n* = 6. (**N**) β-alanine increases *AARS2* GOE mice litter size. Effects of intraperitoneally injected β-alanine on the cumulative number of pups per mother of C57 and *AARS2* GOE mice were measured, *n* = 6. **O** β-alanine reserves primordial follicles and decreases developed follicles of *AARS2* GOE mice. The follicle number of C57 and *AARS2* GOE mice with or without intraperitoneal β-alanine injection was counted at PD3-, 5-, and 7-weeks, *n* = 6. See also Figs. S7 and S8.

We investigated whether inhibiting lactate signal would relieve/prevent POI in mice. β-alanine, a metabolite that is used to alleviate fatigue of muscles during exercise [59], was used to inhibit lactylation [38] and lactate signal. As expected, β-alanine inactivated lactyltransferase activities of both AARS2 and R199C (Fig. 7A), as well as decreased PDHA1 and CPT2 lactylation (Fig. S8A, B) and lactate content (Fig. S8G), increased PDC and CPT2 activity (Fig. S8C, D), OCR level (Fig. S8E), and ATP content (Fig. S8F).

β-alanine inhibited the mTORC1 signaling and proliferation rate induced by AARS2 or R199C overexpression in COV434 cells, and the β-alanine effects was only observed in COV434 cells and not in *AARS2*^*−/−*^ COV434 cells (Fig. 7B–E). Recapturing these observations, β-alanine treatment reversed both mTORC1 and FOXL2 signaling in GCs/ovaries of *AARS2* GOE mice, and these inhibitions were observed in ovaries of C57 mice but not in ovaries of *Aars2* GKO mice (Figs. 7F–K, and S8H, I). These results suggest that inhibiting lactate signal suppresses the signaling pathways activated in POI.

*AARS2* GOE mice intraperitoneally injected with β-alanine exhibited extended estrus (Fig. 7L–M), had increased litter size (Fig. 7N), and had increased primordial follicles and decreased primary, Type4-Type7 follicles (Figs. 7O and S8J). Considering that β-alanine is commonly used as a sports supplement to improve athletic performance [59], it holds promise as a drug for POI prevention and treatment by potentially reducing lactate signaling.

## Discussion

The ovary contains oocytes within primordial follicles, which are fixed in number at birth. Therefore, a faster primordial follicle development rate results in faster follicle depletion, ultimately causing POI. The present study revealed that lactate signal drives follicle development through both promoting GC proliferation and follicle development initiation. Follicle development starts from GC proliferation, which is activated by FSH and sustained by anabolism. Lactate signal, which is exerted through protein lactylation, acts on both GC proliferation and follicle development initiation. AARS2-mediated CPT2 lactylation and inactivation result in FFA accumulation and PPARγ activation, which potentiates FSH to start primordial follicles development. Moreover, the AARS2-catalyzed PDHA1 lactylation and inactivation direct the glycolytic/PPP anabolites to anabolism by inhibiting their oxidation through the TCA cycle [38], and consequently activates such proliferating signals as mTORC1 signaling in GCs to facilitate GC proliferation to begin follicle development. The lactate signal-facilitated GC proliferation and FSH action function in synergy to drive primordial follicles development, and hyper-lactylation causes POI. Previous studies have demonstrated that pyruvate, a product of GC glycolysis, is transferred to oocytes and serves as the primary energy substrate for oxidative metabolism, enabling oocytes to complete meiotic maturation [60]. It is plausible that lactate produced in GCs is transported to oocytes via gap junctions, which are specialized structures that facilitate the direct transfer of small molecules between oocytes and GCs. The lactate transported from granulosa cells to oocytes may contribute to the meiotic maturation defects observed in oocytes of GOE mice (see Fig. 6A).

The hyper-lactylation-driven POI hypothesis is supported by that elevated lactate and FFA observed in POI patients, and by that POI-causing AARS2 mutants gained lactyltransferase activities. Moreover, the causal roles of hyper-lactylation for POI were verified in mouse models. We showed that loss of AARS2 delays and AARS2 overexpression speed up mouse primordial follicles development and follicles depletion. Additionally, inhibiting lactylation with AARS2 inhibitor β-alanine slowed POI onset in mice. As for considering the clinical translation of β-alanine for the prevention or treatment of POI, the dosage, long-term effects on the reproductive system, and potential interactions with other medications need to be thoroughly investigated before considering clinical translation.

We showed that altering AARS2 activity alone is sufficient to affect POI outcome in mice, suggest AARS2 is a promising intervening target of POI. Moreover, AARS2-mediated lactylation potentiates FSH actions to initiate primordial follicles development, suggest AARS2 intervening together with hormonal treatments, may achieve better outcomes in treating POI. Furthermore, as lactate signal exists in all development processes, the mechanism we reveal in the current study may shed light on understanding other development processes and other development disorders than POI.

## Materials and methods

### Clinical samples

Clinical blood samples of 42 POI patients and matched 61 healthy subjects were obtained from volunteers of Obstetrics and Gynecology Hospital of Fudan University, Shanghai, with known age, FSH, LH, AMH, and blood FFA levels. The study was approved by the Human Investigation Ethics Committee of Obstetrics and Gynecology Hospital. With a full understanding of the study, each participant signed the informed consent form voluntarily.

For detection of lactate concentration in sera samples, 800 μL prechilled methanol was added to 200 μL sera to extract metabolites. Supernatants were collected after 12,000 × *g* centrifugation at 4 °C for 10 min, the extraction was repeated twice, and the supernatants were combined, and lyophilized after vacuum dry to remove methanol. The extracted metabolites were redissolved in 600 μL phosphate buffer (0.15 M, pH7.4), centrifuged at 16,099 × *g* (4 °C) for 10 min, and then 550 μL supernatant was transferred into NMR tubes for analysis. All the one-dimensional 1HNMR spectra were obtained at 298 K on a Bruker Advance III 600 MHz NMR spectrometer (600.13 MHz for proton frequency) equipped with an inverse cryogenic probe (Bruker Biospin) using the first increment of the gradient-selected NOESY pulse sequence (NOESYGPPR1D: recycle delay-G1-900-T1-900-tm-G2-900-acquisition). Sixty-four transients were collected into 32 k data points with a spectral width of 20 ppm for each sample and a total relaxation delay of 26 s. All NMR spectra were processed using the spectral width of 20 ppm for each sample and the software package TOPSPIN (V3.6.0 software package TOPSPIN) (Bruker Biospin, Karlsruhe, Germany). For 1H NMR spectra, an exponential window function was employed with a line broadening factor of 1 Hz and zero Hz and zero-filled to 128 K prior to Fourier transformation. Each spectrum was then filled to 128 K prior to Fourier transformation. The characteristic and least- overlapping NMR signals (lactate: δ 4.11) were used to calculate absolute concentration of lactate with the known concentration of TSP.

For analyzing the correlations of lactate/FFA and AMH in POI patients, Blood FFA and AMH concentrations were obtained from the biochemical and endocrine test reports in Obstetrics and Gynecology Hospital. Lactate/FFA concentrations and matched AMH concentrations were plotted using GraphPad Prism 8 software, the correlation between lactate/FFA and AMH was analyzed using linear regression and correlation analysis by GraphPad Prism 8 software.

### Cell lines

The COV434 cell line (immortalized granulosa cells, female) and HEK293T cell line (human embryonic kidney cell lines, female) were maintained in RPMI 1640 (Corning) and Dulbecco’s Modified Eagle’s Medium (DMEM; Gibco), respectively. Both media were supplemented with 10% fetal bovine serum (FBS; Gibco), 100 units/mL penicillin (Invitrogen), and 100 μg/mL streptomycin (Invitrogen). CRISPR/Cas9 system was employed to generate *AARS2*^−/−^ COV434 cells. Briefly, guide RNAs targeting the human AARS2 gene were synthesized, annealed, and cloned into the PX459 vector. The recombinant plasmids were then transfected into COV434 cells using Lipofectamine 3000 (Invitrogen) for 48 h. Transfected cells were selected with 1 μg/mL puromycin (Sangon) and subsequently validated through DNA sequencing and Western blotting. PDHA1/CPT2 KD COV434 cell lines were generated with shRNA-mediated gene silencing. The PLKO vectors containing shPDHA1, shCPT2, and Scramble control sequences were constructed through molecular cloning. Lentivirus particles were produced by cotransfecting HEK293T cells with the respective PLKO vectors along with the packaging plasmids pMD2.G, psPAX2 using Liopfectamine 3000 (Invitrogen). Viral supernatants were harvested 48 h after transfection, filtered through 0.45-μm membranes (Millipore), and stored at −80 °C. For lentiviral transduction, COV434 cells were incubated with viral particles in RPMI 1640 medium containing 10% FBS and 5 μg/mL polybrene (Sigma-Aldrich) for 48 h, followed by selection with 1 μg/mL puromycin (Sangon).

### Mouse primary GCs culture

For the isolation of mouse ovarian primary GCs, the ovaries were aseptically dissected and transferred into a 35-mm culture dish containing 3 mL DMEM/F12 medium (Gibco) supplemented with 10% FBS(Gibco), 100 units/mL penicillin (Invitrogen), and 100 μg/mL streptomycin (Invitrogen). The ovarian tissues were then mechanically dissociated by repeated puncturing using a 1 mL syringe needle. The resulting cell suspension was collected and filtered through a 40 μm cell strainer to remove tissue debris, followed by centrifugation at 1000 rpm for 4 min at room temperature. After centrifugation, the cell pellet was resuspended in 3 mL of fresh DMEM/F12 medium and transferred to a new 35-mm culture dish for incubation. Following 24 h culture, the adherent GCs were washed twice with PBS to remove non-adherent cells and cellular debris. The culture medium was then replaced with fresh DMEM/F12 medium for subsequent experiments.

### Mouse oocytes culture

Wildtype C57 and *AARS2* GOE mice age at postnatal 21 days were intraperitoneal injected with 1 IU/mL pregnant mare’s serum gonadotropin (PMSG) for 48 h and then sacrificed by decapitation. Ovaries were dissected free of fat and connective tissue in M2 medium (Sigma-Aldrich). Under stereomicroscopic observation, the ovaries were mechanically punctured using a 1 mL syringe needle to release cumulus-oocyte complexes. Oocytes at Germinal vesicle (GV) stage were collected using a mouth-controlled aspiration system equipped with a finely pulled glass Pasteur pipette and maintained in M2 medium (Sigma-Aldrich). For subsequent culture, the oocytes were transferred to M16 medium (Sigma-Aldrich) and incubated under mineral oil at 37 °C in a humidified atmosphere containing 5% CO_2_.

### Mouse follicle culture

Ovaries from 16- to 20-day-old *WT*, *AARS2* GOE, and *Aars2* GKO mice were cut into 4 pieces and enzymatically digested in α-MEM medium containing 0.1% collagenase I (Sigma-Aldrich, USA) and 0.02% DNase I (Invitrogen) at 37 °C with 5% CO_2_ for 30 min. Following enzymatic digestion, primary follicles were mechanically isolated in L15 medium supplemented with 1% FBS. Individual follicles were then encapsulated in 0.5% (w/v) alginate hydrogel as previously described [61]. The alginate-encapsulated follicles were cultured 24-well plates, with each well containing 500 μL of α-MEM medium supplemented with 10 mIU/mL recombinant FSH, 3 mg/mL bovine fetuin, 5 μg/mL transferrin, and 5 ng/mL disodium selenite. The cultures were maintained for 4 days at 37 °C with 5% CO_2_, with half of the medium replaced every 48 h. After the 4-day culture period, the alginate-encapsulated follicles were transferred to L15 medium containing 10 U/mL alginate lyase and incubated at 37 °C for 20 min to dissolve the alginate matrix. Finally, the released follicles were collected and fixed in 4% PFA for subsequent analysis.

### Mouse Lines

All animal procedures were in accordance with the animal care committee at Fudan University. The hAARS2-transgenic mice (*Rosa26*^*AARS2/AARS2*^) were generated on a C57BL/6 genetic background using CRISPR-Cas9-mediated genome editing technology to insert CAG-LSL-hAARS2-WPRE-PA cassette into the *Rosa26* locus. Transgenic mice carrying AMH *receptor type 2 (Amhr2)* and *zona protein 3(ZP3)* promoter-mediated Cre recombinase, which are specifically expressed in GCs and oocytes, respectively, were kindly gifts of Prof. Chao-Jun Li, Nanjing Medical University. The *Aars2* CKO mice (*Aars2*^flox/flox^ mice containing two loxP sites flanking exons 1-5) were generated by employing Cre-LoxP recombination technology. For tissue-specific overexpression models, *Rosa26*^*AARS2/AARS2*^ mice were crossed with *Amhr2*^*Cre*^ and *ZP3*^*Cre*^ mice to produce *Amhr2*^*Cre*^*; Rosa26*^*AARS2/WT*^ (*AARS2* GOE) and *ZP3*^*Cre*^*; Rosa26*^*AARS2/WT*^ (*AARS2* ZOE) mice, which specifically overexpress hAARS2 in GCs and oocytes, respectively. For the GC-specific knockout model, *Aars2*^*loxp/loxp*^ mice were backcrossed with *Amhr2*^*Cre*^ mice for 2 generations to obtain *Amhr2*^*Cre*^*; Aars2*^*loxp/loxp*^ (*Aars2* GKO) mice, which exhibit specific deletion of *Aars2* in GCs.

### Mouse ovarian FFA quantification

FFA quantification in mouse ovaries was performed using a commercial Free Fatty Acid Assay Kit (Abcam) following the manufacturer’s instructions. Briefly, wildtype C57, *AARS2* GOE, and *Aars2* GKO mice aged at 5- to 12-weeks were sacrificed during the diestrus phase of estrous cycle. Ovaries were immediately collected, weighted, and processed for analysis. For lipid extraction, Ovarian tissues were homogenized in 200 μL of chloroform/Triton X-100 mixture (1:1, v/v) using a tissue homogenizer. The homogenates were then vacuum-dried to remove organic solvents. The resulting lipid extracts were reconstituted in 200 μL of assay buffer and incubated with the reaction mixture at room temperature for 30 min. Absorbance was measured at 570 nm using a SpectraMax i3x Multi-Mode Plate Reader (Molecular devices). FFA content was determined using a standard curve generated from serial dilutions of provided FFA standards. The final FFA concentrations were normalized to ovarian weight and expressed as nmol/mg tissue.

### FSH and E2 detection

Whole blood samples were collected from 12- to 20-week-old *WT* and *AARS2* GOE mice during the proestrus stage. Serum was isolated through gradient centrifugation at 3000 rpm for 15 min at 4 °C. FSH and E2 levels were measured using commercial ELISA kits (FSH ELISA kit and E2 ELISA Kit, ABclonal) according to the manufacturer’s protocols. For in vitro E2 production analysis, COV434 cells were seeded at a density of 1 × 10^5^ cells/mL and stimulated with 100 ng/mL recombinant FSH for 24 h to induce E2 synthesis. After incubation, 100 μL of conditioned medium was collected for E2 measurement using the E2 ELISA kit. All measurements were performed in triplicate, and hormone concentrations were determined based on standard curves generated from the respective assay kits.

### ATP assay

ATP content of COV434 cell lines and mouse primary GCs was measured using CellTiter-Glo 2.0 Cell Viability Assay Kit (Promega) according to the manufacturer’s protocol. Briefly, cells were seeded in opaque-walled 96-well plates at a density of 10000 cells per well in 100 μL of culture medium. Following cell attachment, an equal volume (100 μL) of CellTiter-Glo2.0 reagent was added to each well, and the plates were incubated at room temperature for 10 min to allow cell lysis and stabilization of the luminescent signal. Luminescence intensity was measured using a SpectraMax i3x Multi-Mode Plate Reader (Molecular Devices). All measurements were performed in triplicate, and ATP levels were normalized to cell number.

### CPT2 activity

Detection of CPT2 activity was performed as previously described [62]. Briefly, pCMV-CPT2-Flag was co-transfected into *WT* and *AARS2*^*−/−*^ COV434 cells, putting back AARS2 and R199C-HA. CPT2-Flag protein was immunoprecipitated using ANTI-FLAG M2 Affinity Gel (Sigma-Aldrich) after 48 h of transfection. The CPT2 enzymatic assay was performed in a 1.5 mL reaction system containing 5 mM Tris-Hcl (pH 8.0), 12 mM KCl, 1 mM EDTA (pH 8.0), 2 mM CoA (Beyotime), and 2 mM palmitoyl-L-carnitine (Sigma-Aldrich). The reaction was initiated by adding the purified CPT2 protein and incubared at 37 °C with constant shaking for 10 min. Subsequently, 50 μL of reaction mixture supernatant was transferred to DTNB buffer, and OD_410_ was immediately measured using a Spectramax i3x Multi-Mode Plate Reader (Molecular Devices). CPT2 activity was calculated as [OD_410_(0 min)-OD_410_(10 min)]/10 min. All measurements were performed in triplicate, and enzyme activity was normalized to protein concentration.

### PDC activity

The PDH Enzyme Activity Microplate Assay kit (Abcam) was used to measure PDC activity of transfected COV434 cells. Cells were homogenized to determine protein concentrations by BCA Protein Assay Kit (Beyotime) and supernatants were used for PDH assay according to the manufacturer’s protocol.

### Cell proliferation

The proliferative capacity of COV434 cells and mouse primary GCs was evaluated using the Cell Counting Kit-8 (CCK-8; YEASEN). COV434 cells with different treatment were trypsinized and seeded in 96-well plates at a density of 1000 cells per well in 100 μL of culture medium, with 4 replicates for each condition. After cell attachment, 10 μL CCK-8 solution was added to each well, and the plates were incubated at 37 °C for 2 h, OD_450_ was measured daily at the same time point using a Spectramax i3x Multi-Mode Plate Reader (Molecular Devices). For mouse primary GCs, cells were plated at a density of 1000 cells per well in 96-well plates and cultured for 7 days. The CCK-8 assay was performed daily by adding 10 μL of CCK-8 solution to each well, followed by a 2 h incubation at 37 °C. OD_450_ was measured using the same microplate reader. Cell proliferation curves were generated by plotting the absorbance values against time.

### PDHA1/CPT2 lactylation assay

For detection of PDHA1 and CPT2 lactylation levels in COV434 cells ex vivo, *WT* and *AARS2*^−/−^ COV434 cells expressing either wildtype and mutant AARS2 were co-transfected with pcDNA3.1-PDHA1-Flag or pcDNA3.1-CPT2-Flag constructs using Lipofectamine 3000 (Invitrogen) according to the manufacturer’s instruction. After 48 h of transfection, whole cell lysates were obtained using 0.1% NP-40 buffer supplemented with protease and phosphatase inhibitor cocktail (Sigma-Aldrich). PDHA1-Flag and CPT2-Flag proteins were immunoprecipitated using ANTI-FLAG M2 Affinity Gel (Sigma-Aldrich). Lactylation levels at specific lysine residues (PDHA1-K336 and CPT2-K457/8) were detected using homemade PDHA1^Lac-K336^ and CPT2^Lac-K457/8^ antibodies through Western blot analysis. For in vitro lactylation analysis, synthetic peptides containing PDHA1-K336 and CPT2-K457/8 residues, as well as immunoprecipitated recombinant PDHA1 and CPT2 proteins, were subjected to lactylation reactions. Peptides were eluted and immobilized on nitrocellulose filter membranes, while recombinant proteins were solubilized in SDS buffer. Lactylation levels were subsequently detected using the PDHA1^Lac-K336^ and CPT2^Lac-K457/8^ antibodies.

### In vitro lactylation

In vitro lactylation reactions of synthetic substrate peptides were carried out in a 30 μL reaction mixture containing 6.7 U/mL pyrophosphatase (Sigma-Aldrich), 50 mM HEPES (pH 7.5), 25 mM KCl, 2 mM MgCl_2_, 15 mM lactic acid (Sigma-Aldrich), 4 mM ATP, 100 nM recombinant AARS2-WT or AARS2-R199C protein, and 0.05 mg/mL synthetic peptides (PDHA1: Ac-MVNSNLASVEELKEIDVEVR; CPT2: Ac-EFLKKQKLS). The reaction mixture was incubated at 37 °C for 12 h. Following incubation, the peptides were desalted using C18 ZipTips (Millipore) and immobilized on nitrocellulose filter membranes for subsequent analysis. For lactylation of recombinant PDHA1 and CPT2 proteins, 5 μg pcDNA3.1-PDHA1-Flag, pCMV-CPT2-Flag were transfected to HEK293T cells for 48 h. Recombinant proteins were immunoprecipitated using ANTI-FLAG M2 Affinity Gel (Sigma-Aldrich) and resuspended in 90 μL of lactylation reaction buffer containing the same concentrations of HEPES, KCl, MgCl_2_, lactic acid, ATP as described above. The reaction was carried out at 37 °C for 3 h. All reactions were performed in triplicate, and lactylation levels were quantified using Western blot analysis with specific antibodies.

### Targeted metabolic analysis with LC-MS/MS

Metabolites from transfected COV434 cells, *WT*, *AARS2* GOE mice GCs were extracted by adding 800 μL prechilled extraction buffer (acetonitrile: methanol: H_2_O = 2:2:1, v/v/v). Supernatants were collected after 12,000 × *g* centrifugation at 4 °C for 10 min. The extracted metabolites were then subjected to targeted metabolomic analysis using an AB SCIEX QTRAP 6500+ liquid chromatography-tandem mass spectrometry (LC-MS/MS) system (AB SCIEX). Specifically, metabolites associated with glycolysis, the PPP, and the TCA cycle were quantified. Data acquisition and analysis were performed using Analyst 1.7 software (AB SCIEX), with metabolite identification based on retention time and mass-to-charge ratio (m/z) matching against authentic standards. All samples were analyzed in triplicate, and metabolite levels were normalized to cell numbers.

### Immunofluorescence and confocal microscopy

For ovarian immunofluorescence staining, the paraffin sections were dewaxed, rehydrated, and boiled in citrate buffer for antigen retrieval. The sections were permeabilized with 0.1% Triton X-100 and 0.1% Tween20, followed by blocking with 5% goat serum for 1 h at room temperature. Primary antibodies against FOXL2, MVH, PPARγ, MCT1, and MCT4 were applied and incubated overnight at 4 °C. After three washes with PBS, sections were incubated with Alexa Fluor^TM^ 488-conjugated goat anti-rabbit IgG (H + L) highly cross-adsorbed secondary antibody (Invitrogen) for 2 h at room temperature, followed by counterstaining with DAPI for 5 min. Slides were mounted and imaged using a Nikon A1 confocal microscope (Nikon).

For oocytes immunofluorescence staining, in vitro cultured oocytes from *WT* and *AARS2* GOE mice at 12 h and 16 h time points were fixed in 4% PFA for 30 min. Oocytes were permeabilized with 0.05% Triton X-100 for 30 min and blocked with 3% BSA for 1 h at room temperature. Primary anti-α-tubulin antibody (1:100, Abcam) was applied and incubated overnight at 4 °C. After washing in 3-5 drops of PBS, oocytes were incubated with Alexa Fluor^TM^ 488-conjugated goat anti-rabbit IgG (H + L) highly cross-adsorbed secondary antibody (Invitrogen) for 2 h and counterstained with DAPI for 5 min at room temperature. Imaging was performed using a Nikon A1 confocal microscope (Nikon).

For immunofluorescence staining of in vitro cultured follicles, fixed follicles were dehydrated in 30% sucrose and permeabilized/blocked in a solution containing 10% Triton X-100 and 5% goat serum for 24 h. Follicles were incubated with primary antibodies against FOXL2 at 4 °C for 48 h, washed in PBS, and then incubated with Alexa Fluor^TM^ 546-conjugated goat anti-Rabbit IgG(H + L) highly cross-adsorbed secondary antibody (Invitrogen) and DAPI at 4 °C for 24 h. For follicles larger than 100 μm, a modified Clear Unobstructed Brain Imaging Cocktails and Computational analysis (CUBIC) tissue clearing method was employed. Stained follicles were incubated in CUBIC-1 buffer (25% urea, 25% NNNN-Tetrakis ethylenediamine, 15% Triton X-100) for 10 days, 1/2 CUBIC-2 buffer (12.5% urea, 25% sucrose, 5% Triethanolamine) for 24 h, and CUBIC-2 (25% urea, 50% sucrose, 10% Triethanolamine) buffer for 24 h at 37 °C. Cleared follicles were embedded in a 35-mm glass-bottom dish containing fresh clearing solution and imaged using a ZEISS LSM 880 confocal microscope (ZEISS).

### Plasmids and transfection

The coding sequences (CDS) of AARS2, PDHA1, and CPT2 were amplified from HEK293T cDNA and subsequently cloned into expression vectors using restriction enzyme sites. Specifically, the CDS regions were inserted into Xho I and EcoR I sites of the pcDNA3.1-Flag vector, the BamH I and EcoR I sites of the pCMV-Flag vector, or the EcoR I and Xba I restriction sites of the PCDH-Flag/HA vector. Cloning was performed using the CloneExpress MultiS OneStep Cloning Kit (Vazyme) following the manufacturer’s protocol. Site-directed mutagenesis to generate AARS2 mutants was carried out using the Mut Express MultiS Fast Mutagenesis kit (Vazyme) according to the manufacturer’s instructions. All constructed plasmids were verified by Sanger sequencing. For transfection, plasmids were introduced into cells using Lipofectamine 3000 (Invitrogen) according to the manufacturer’s instructions.

### Estrous cycle and fertility assays

To asses reproductive cyclicity, vaginal smears were collected from *WT*, *AARS2* GOE and *Aars2* GKO female mice over 15 consecutive days strating at 8 weeks of age. Smears were stained with crystal violet, and the estrous cycle stage was determined by vaginal cytology according to a previously established protocol [63]. To evaluate reproductive competency, *WT*, *AARS2* GOE, and *AARS2* GKO female mice born at the same time at age of 4 weeks were paired with wildtype male mice of proven fertility at 8 weeks of age. Mating pairs were maintained until the females reached 40 weeks of age. Litter size and inter-litter interval were recorded continuously throughout the study period. To control for potential effects of male aging, male mice were replaced with new 8-weeks-old males after 20 weeks of mating. All reproductive parameters, including the number of litters, litter size, and intervals between litters, were analyzed to determine the impact of AARS2 manipulation on female fertility.

### Quantification of follicle

To compare the size of primordial follicle pool among *WT*, *AARS2* GOE and *Aars2* GKO mice, ovaries of each genotype at PD1 were obtained under a stereomicroscope, fixed in 4% paraformaldehyde, dehydrated, and embedded in paraffin and then cut into 5-μm sections continuously, immunofluorescence stained with MVH for one of every seven sections. The number of MVH-positive follicles was counted using a Nikon A1 confocal microscope (Nikon), and the total number of oocytes was estimated by multiplying the counted number by 7. For assessment of GC proliferation, ovaries of mice of each genotype or treatment with lactate, β-alanine, THI, GW9662 were embedded in paraffin and sectioned at 5-μm thickness. Every 6 sections was immunofluorescence stained for FOXL2. The total number of secondary follicles (defined as follicles with two or more layers of GCs) in the stained sections was quantified using a Nikon A1confocal microscope. As for quantifying the maximum cross-sectional area of in-vitro cultured follicles, follicles cultured for 4 days were immunofluorescence stained with FOXL2, maximum cross-section of every follicle was obtained by ZEISS 880 confocal microscope (ZEISS), and the area was quantified by Aivia software (Leica) based on FOXL2 signaling in follicles.

Quantification of ovarian follicles was performed as previously described [10]. Briefly, ovaries were fixed in 4% paraformaldehyde, dehydrated, and paraffin-embedded. serially 5-μm sections were stained with hematoxylin and eosin (H&E) for morphological analysis. Follicles at different developmental stages (primordial, type 3, type 4, type 5, type 6, type 7) were counted in all sections of each ovary, based on the well-accepted standards established by Pedersen and Peters [64]. Only follicles containing oocytes with clearly visible nuclei were scored, as previously reported [10].

### Lipid droplets counting

For counting lipid droplets in GCs, frozen ovarian sections were stained with oil red O to visualize neutral lipids, followed by counterstaining with hematoxylin to label nuclei. Lipid droplets within GCs of secondary or more advanced-stage follicles were identified and counted using Image J software.

### Oxygen consumption rate (OCR)

OCR of COV434 cells and mouse primary GCs were measured using the Oxygraph-2k (O2k, OROBOROS INSTRUMENTS) following the manufacturer’s protocol. Briefly, the O2k chambers were filled with 3.3 mL of PBS and stabilized for 30 min to achieve a consistent oxygen baseline. Cells were detached using 0.05% trypsin, resuspended in PBS, and adjusted to a concentration of 1 × 10^6^ cells/mL. A 200 μL aliquot of the cell suspension was added to each chamber, and the system was allowed to stabilize for 5 min. To assess mitochondrial respiration, 2.5 μM oligomycin was injected into the chambers. OCR was calculated as the difference between the O_2_ consumption slope before and after oligomycin addition. All measurements were performed in triplicate, and data were normalized to cell number.

### Cell treatment with lactate, β-alanine, SO, α-CHCA, and GW9662

30 mM methyl L-lactate, 10 mM SO, 5 mM α-CHCA and 30 mM β-alanine were added to COV434 cells for 3 h to mimic the enhanced and attenuated lactate signaling. In the in vitro culture system, *WT* and *AARS2* GOE follicles were treated with sequential doses of 10 and 50 mIU FSH in the presence of either DMSO or 10 μM GW9662 for 4 days. This experimental setup was used to evaluate the role of PPARγ in mediating FSH signaling during follicular development. To investigate the effects of lactate signaling on follicular development, *WT* and *Aars2* GKO follicles were cultured for 4 days in medium supplemented with10 mIU FSH and either PBS or 30 mM methyl L-lactate. Follicle growth and development were monitored to assess the impact of lactate signaling on folliculogenesis.

### Isothermal titration calorimeter

Isothermal titration calorimeter (Microcal) was used to compare the binding affinity of lactate to R199C and AARS2 according to the manufacturer’s instructions. Briefly, 80 μM purified AARS2 or R199C protein, suspended in Tris-HCl buffer (pH7.4), was loaded into the sample cell, while 2 mM lactate (pH7.4) was loaded into the injection syringe. A total of 20 injections were performed: the first injection consisted of 0.5 μL over 1 s, followed by a 120-s spacing and a 5-s filter period; the subsequent 19 injections consisted of 2 μL over 4 s, with the same spacing and filter period. The heat released upon each injection was recorded by the instrument. Data were analyzed using Origin 7.0 software to generate binding curves and calculate the binding parameters, including the number of binding sites (N), binding affinity (K), enthalpy change (ΔH), and entropy change (ΔS). The parameters of lactate binding to AARS2 were as follows: N (1.28 ± 0.086 sites), K (8.23–17.86 μM), ΔH (−3.439E4 ± 3131 cal/mol), ΔS (−92.7 cal/mol/deg). For lactate binding to the R199C mutant, the parameters were as follows: N (0.619 ± 0.0147 sites), K (2.6–3.7 μM), ΔH (-5.428E4 ± 1759 cal/mol), ΔS (−156.9 cal/mol/deg).

### Expression and purification of AARS2-WT and R199C

The PET28b-AARS2 plasmid, with a 30-base pair deletion in the N-terminal signaling sequence, was constructed using gene cloning techniques. Additionally, the PET28b-AARS2-R199C mutant plasmid was generated using the Mut Express MultiS Fast Mutagenesis Kit (Vazyme) according to the manufacturer’s instructions. Both plasmids were transformed into *E. coli* BL21 competent cells for protein expression. The transformed BL21 cells were cultured in LB medium at 37 °C with shaking until the optical density at 600 nm (OD_600_) reached 0.5. Protein expression was induced by adding 0.2 mM IPTG (Sangon), followed by incubation at 18 °C for 16 h. Cells were harvested by centrifugation and lysed via sonication on ice. The lysates were clarified by centrifugation, and the supernatant was loaded onto an AKTA purification system (cytiva). The AARS2-6xHis and AARS2-R199C-6xHis fusion proteins were purified using a nickel-nitrilotriacetic acid (Ni-NTA) column, followed by buffer exchange and concentration. Protein purity was verified by SDS-PAGE, and concentration was determined using a Bradford assay.

### Structural modeling

Structural modeling of human AARS2 was performed using the alanyl-tRNA synthetase (AlaRS) structure from *Archaeoglobus fulgidus* (PDB ID: 3WQY_A) as a template [65]. Sequence alignment between human AARS2 (985 aa) and template *A. fulgidus* AlaRS (906 aa) was conducted, followed by homology modeling using the SWISS-MODEL server. The resulting model was refined and validated for structural accuracy. Docking of the *A. fulgidus* tRNA^Ala^ and alanyl-adenylate into the model was done after superimposition of template chain A with the modeled human mtAlaRS using PyMOL (Schrodinger) software. AARS2 mutations associated with POI, leukodystrophy, and cardiomyopathy were shown as red, violet, and green spheres, respectively, in PyMOL.

### Mice intraperitoneal injection

20 mg/kg sodium lactate with adjusted pH was intraperitoneal injected into *WT*, *Aars2* GKO mice from PD3 days to PD13 days to evaluate the effect of lactate signaling on GCs proliferation and PPARγ activation. For detecting lactate’s effect on FSH signaling, 0.1 IU/mL FSH was intraperitoneal injected to *WT*, *Aars2* GKO mice for 12 h after consecutive lactate injection for 10 days. 10 mg/kg SO was intraperitoneal injected to *WT*, *AARS2* GOE mice from PD3 days to PD15 days to evaluate the effect of eliminated lactate signaling on rescuing *AARS2* GOE phenotype. Similarly, 10 mg/kg THI was intraperitoneal injected to *WT* female mice since PD3 days to PD13 days to evaluated the effect of PPARγ activation on GC proliferation. To study the role of PPARγ inhibition, 10 mg/kg GW9662 was intraperitoneal injected to *WT*, *AARS2* GOE mice from PD3 days to PD15, followed by intraperitoneal injected 0.01 or 0.1 IU/ mL FSH for 12 h to assess its ability to rescue the *AARS2* GOE phenotype. To further explore the effects of lactate signaling modulation, 20 mg/kg β-alanine was intraperitoneal injected into *WT*, *AARS2* GOE mice, and *Aars2* GKO mice. For FOXL2 signaling analysis, β-alanine was intraperitoneal injected to *WT*, *AARS2* GOE mice, and *Aars2* GKO mice from PD3 to PD20. For ovarian morphology and fertility assessment, β-alanine was injected every other day from PD2 weeks to PD8 weeks. Mice were sacrificed at PD3, PD5, PD7, and PD16 weeks to collect ovaries for morphological evaluation, while PD8-week-old mice were mated with age-matched WT males to compare fertility across groups.

### Mitochondrial isolation and lactate detection

A hypotonic buffer containing 20 mM HEPES (pH7.5), 140 mM KCl, 10 mM EDTA, and 5 mM MgCl_2_ with a protease and phosphatase inhibitor cocktail was added to COV434 cells or ovarian homogenate. The cells or homogenate were broken with a syringe and then centrifuged at 800 × *g* for 10 min to separate the nucleus and other debris. The supernatant was then centrifuged at 12,000 × *g* and 4 °C for 30 min. the precipitate was washed thrice with a hypertonic buffer containing 20 mM HEPES (pH 7.5), 800 mM KCl, 10 mM EDTA, 5 mM MgCl2 and thrice with PBS. Mitochondrial were then lysed by ultrasonication on ice. Metabolites were extracted by adding methanol to the lysates to achieve a final concentration of 80% (v/v), followed by centrifugation to remove insoluble material. The supernatant containing mitochondrial metabolites was collected, and lactate levels were quantified using NMR spectroscopy.

### Quantitation and statistical analysis

Unless specified, results are expressed as means ± SD. Two-tailed Student’s *t*-test was performed for the two-group analysis, One-way Welch’s ANOVA to compare more than two groups. Differences were considered statistically significant if the *p* value was less than 0.05.

## Supplementary information

Supplemental materials

Original Western blots

## Data Availability

Any additional information required to reanalyze the data reported in this work paper is available from Wei Xu upon request.
