# Supplementary material for: AARS2-catalyzed lactylation induces follicle development and premature ovarian insufficiency
Source: Cell Death Discov. 2025 Apr 29;11:209. doi: 10.1038/s41420-025-02501-0 (PMC12041370; doi:10.1038/s41420-025-02501-0)
Supplement: Supplementary file 1 — Supplemental materials [file 41420_2025_2501_MOESM1_ESM.pdf]

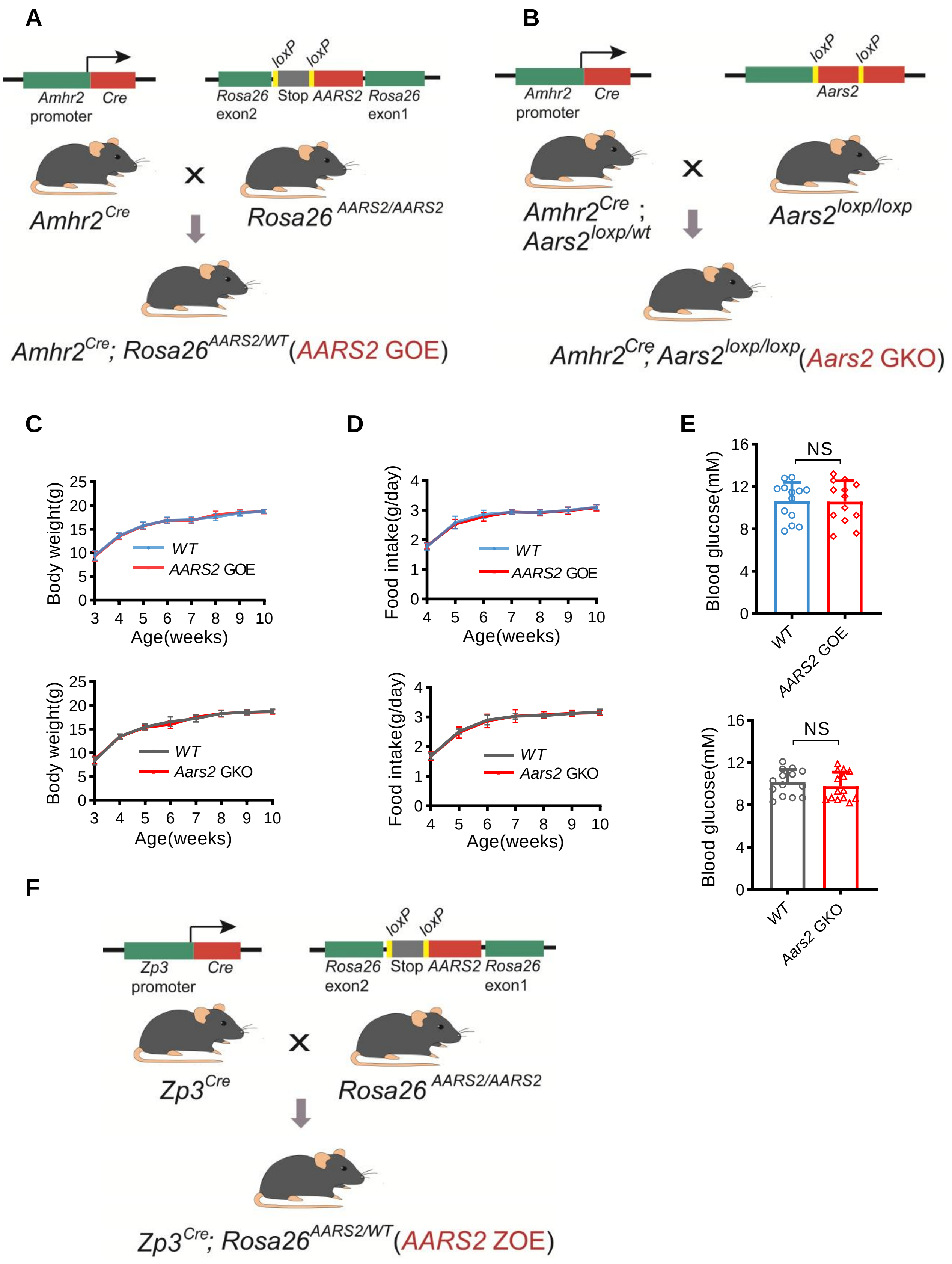

A
loxP
loxP
Amhr2 promoter
Cre
Rosa26 exon2
Stop
AARS2
Rosa26 exon1
Amhr2Cre
Rosa26 AARS2/AARS2
Amhr2Cre; Rosa26AARS2/WT (AARS2 GOE)
B
Amhr2 promoter
Cre
Aars2
Amhr2Cre ; Aars2loxp/wt
Aars2loxp/loxp
Amhr2Cre; Aars2loxp/loxp (Aars2 GKO)
C
Body weight(g)
Age(weeks)
WT
AARS2 GOE
Aars2 GKO
D
Food intake(g/day)
Age(weeks)
WT
AARS2 GOE
Aars2 GKO
E
Blood glucose(mM)
NS
WT
AARS2 GOE
Aars2 GKO
F
Zp3 promoter
Cre
Rosa26 exon2
Stop
AARS2
Rosa26 exon1
Zp3Cre
Rosa26 AARS2/AARS2
Zp3Cre; Rosa26AARS2/WT (AARS2 ZOE)

Supplementary Figure 1. AARS2 has no effect on basal metabolism. Related to Figure 2.

(A) Strategy to generate *AARS2* GOE mice**.** *Rosa26*$^{AARS2/AARS2}$ mice were backcrossed to *Amhr2*^{Cre} mice to obtain *Amhr2*^{Cre}*; Rosa26*$^{AARS2/WT}$ mice specifically overexpress hAARS2 in GCs (*AARS2* GOE).

(B) Strategy to generate *Aars2* GKO mice. *Aars2*$^{loxp/loxp}$ mice were backcrossed to *Amhr2*^{Cre} mice for 2 generations to obtain *Amhr2*^{Cre}*; Aars2*$^{loxp/loxp}$ mice specifically knockout *Aars2* in GCs (*Aars2* GKO).

(C-E) AARS2 has no effect on basal metabolism. Weekly cumulative body weight (**C**, n=14), food intake (**D**, n=6), and random blood glucose (**E**, n=13) in female C57, *AARS2* GOE (top panel) and *Aars2* GKO (bottom panel) mice were detected.

(F) Strategy to generate *AARS2* ZOE mice**.** *Rosa26*$^{AARS2/AARS2}$ mice were backcrossed to *Zp3*^{Cre} mice to obtain *Zp3*^{Cre}*; Rosa26*$^{AARS2/WT}$ mice specifically overexpress hAARS2 in oocytes (*AARS2* ZOE).

(A) Strategy to generate *AARS2* GOE mice**.** *Rosa26*$^{AARS2/AARS2}$ mice were backcrossed to *Amhr2*^{Cre} mice to obtain *Amhr2*^{Cre}*; Rosa26*$^{AARS2/WT}$ mice specifically overexpress hAARS2 in GCs (*AARS2* GOE).

(B) Strategy to generate *Aars2* GKO mice. *Aars2*$^{loxp/loxp}$ mice were backcrossed to *Amhr2*^{Cre} mice for 2 generations to obtain *Amhr2*^{Cre}*; Aars2*$^{loxp/loxp}$ mice specifically knockout *Aars2* in GCs (*Aars2* GKO).

(C-E) AARS2 has no effect on basal metabolism. Weekly cumulative body weight (**C**, n=14), food intake (**D**, n=6), and random blood glucose (**E**, n=13) in female C57, *AARS2* GOE (top panel) and *Aars2* GKO (bottom panel) mice were detected.

(F) Strategy to generate *AARS2* ZOE mice**.** *Rosa26*$^{AARS2/AARS2}$ mice were backcrossed to *Zp3*^{Cre} mice to obtain *Zp3*^{Cre}*; Rosa26*$^{AARS2/WT}$ mice specifically overexpress hAARS2 in oocytes (*AARS2* ZOE).

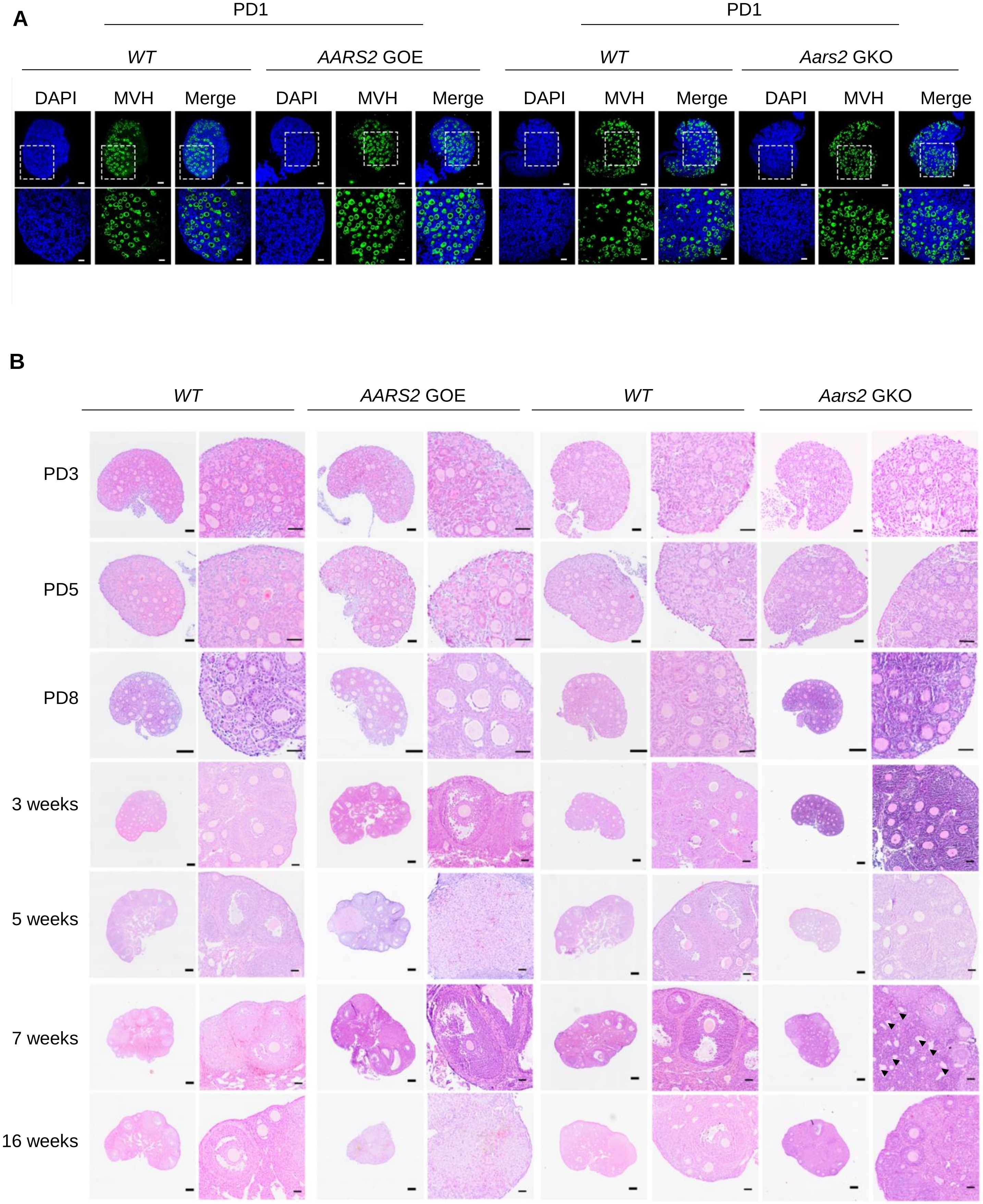
A
PD1
PD1
WT
AARS2 GOE
WT
Aars2 GKO
DAPI
MVH
Merge
B
WT
AARS2 GOE
WT
Aars2 GKO
PD3
PD5
PD8
3 weeks
5 weeks
7 weeks
16 weeks

Supplementary Figure 2. AARS2 promotes primordial follicle development. Related to Figure 2.

(A) AARS2 has no effects on primordial follicle pool size at PD1. Ovarian sections of C57, *AARS2* GOE, and *Aars2* GKO mice were stained with DAPI and oocyte marker MVH (scale bars, 50 μm (top panel), 20 μm (bottom panel)).

(B) AARS2 promotes follicle development. Ovarian morphologies at PD3, PD5, PD8, and 3-, 5-, 7-, 16-week-old *AARS2* GOE, *Aars2* GKO and *WT* mice were detected using H&E staining (scale bars, 50 μm (PD3-5, left panel), 200 μm (PD8-16weeks, left panel), 50 μm (right panel)).

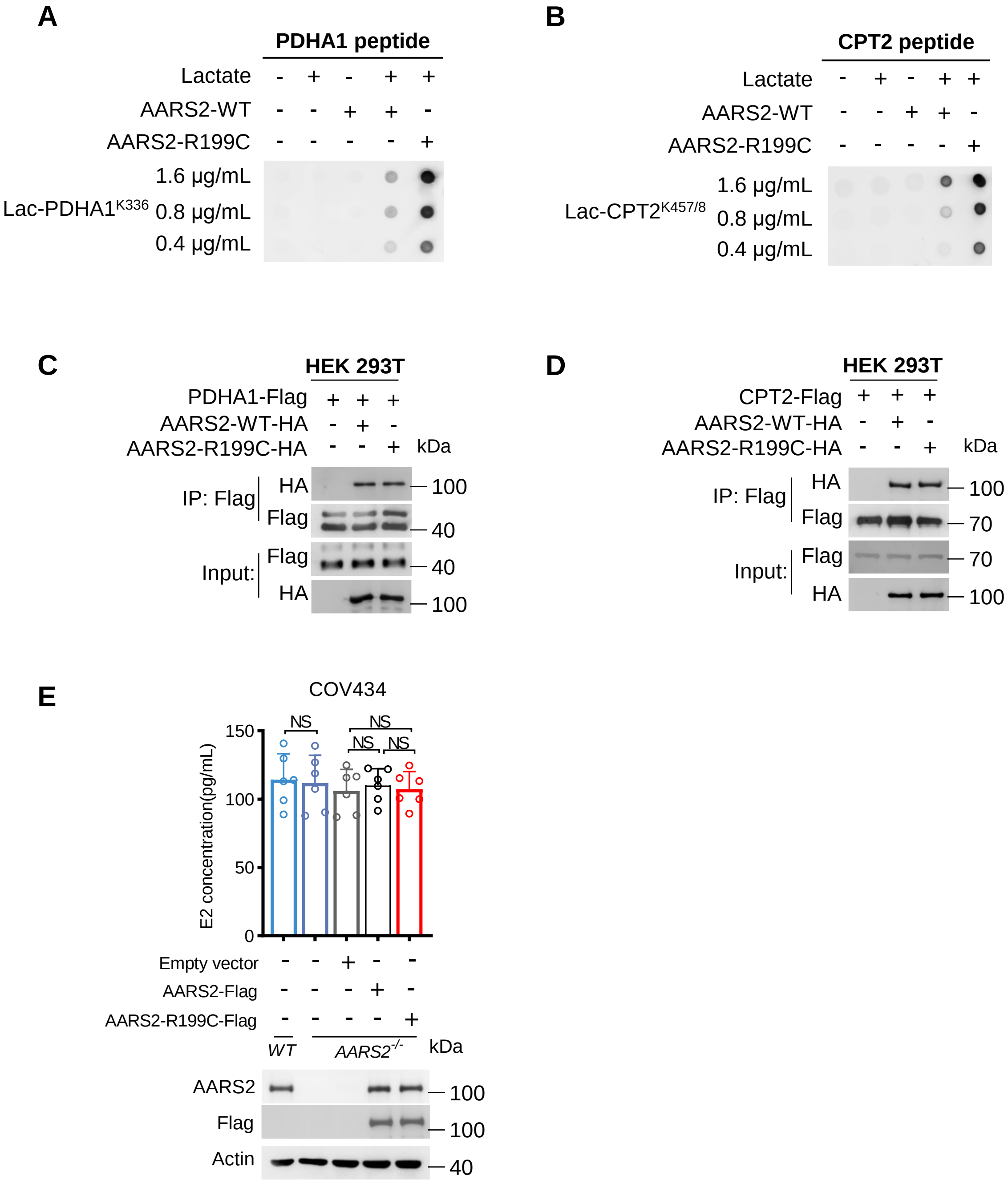

Supplementary Figure 3. R199C has enhanced lactyltransferase activity. Related to Figure 3.

(A-B) R199C has stronger lactyltransferase activity than AARS2. The abilities to form Lac-K336 and Lac-K457/8 in synthetic PDHA1 (**A**) and CPT2 (**B**) peptides were detected *in vitro*.

(C-D) AARS2 and R199C have same binding affinity for substrate protein. The interaction of ectopically expressed AARS2-WT-HA and AARS2-R199C-HA with PDHA1-Flag (**C**) and CPT2-Flag (**D**) in HEK293T were compared.

(E) R199C has no effect on COV434 cells E2 synthesis. E2 concentrations were detected in medium from FSH stimulated COV434, AARS2−/− COV434, and AARS2−/− COV434 that co-expressed AARS2 or R199C cells (n=6).

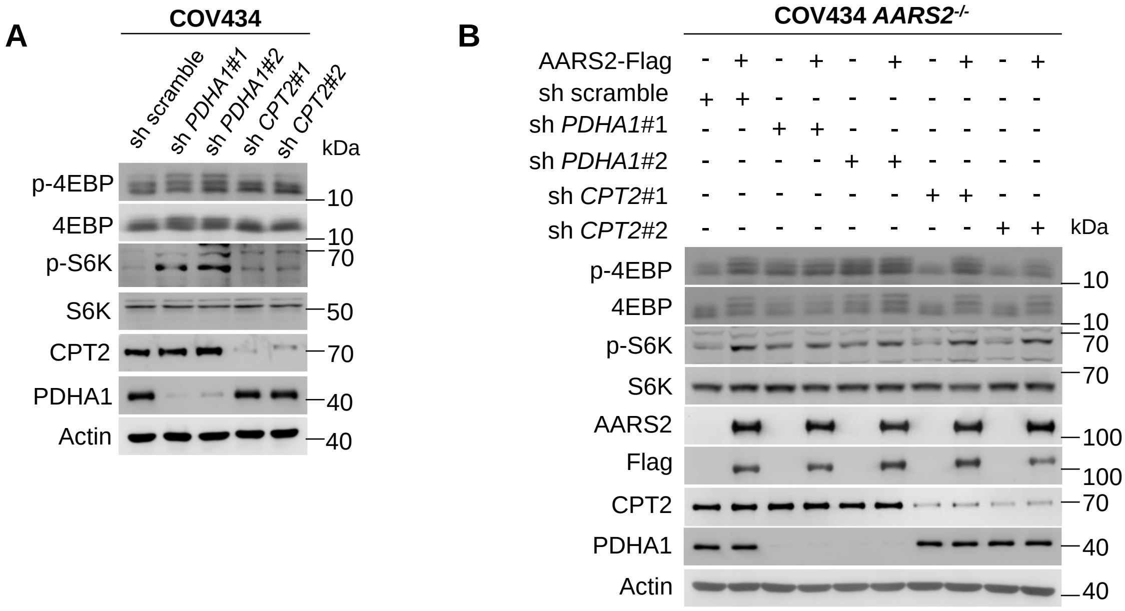

Supplementary Figure 4. AARS2 activates mTORC1 through PDHA1, but not CPT2 inhibition. Related to Figure 4.

(A) PDHA1 inhibition activates mTORC1 in COV434 cells. p-S6K and p-4EBP were detected in PDHA1- and CPT2-silenced COV434 cells.

(B) AARS2 activates mTORC1 in a PDHA1-dependent way. p-S6K and p-4EBP were detected in *AARS2*$^{-/-}$ COV434 cells with PDHA1 or CPT2 silencing, transfected with or without AARS2-Flag.

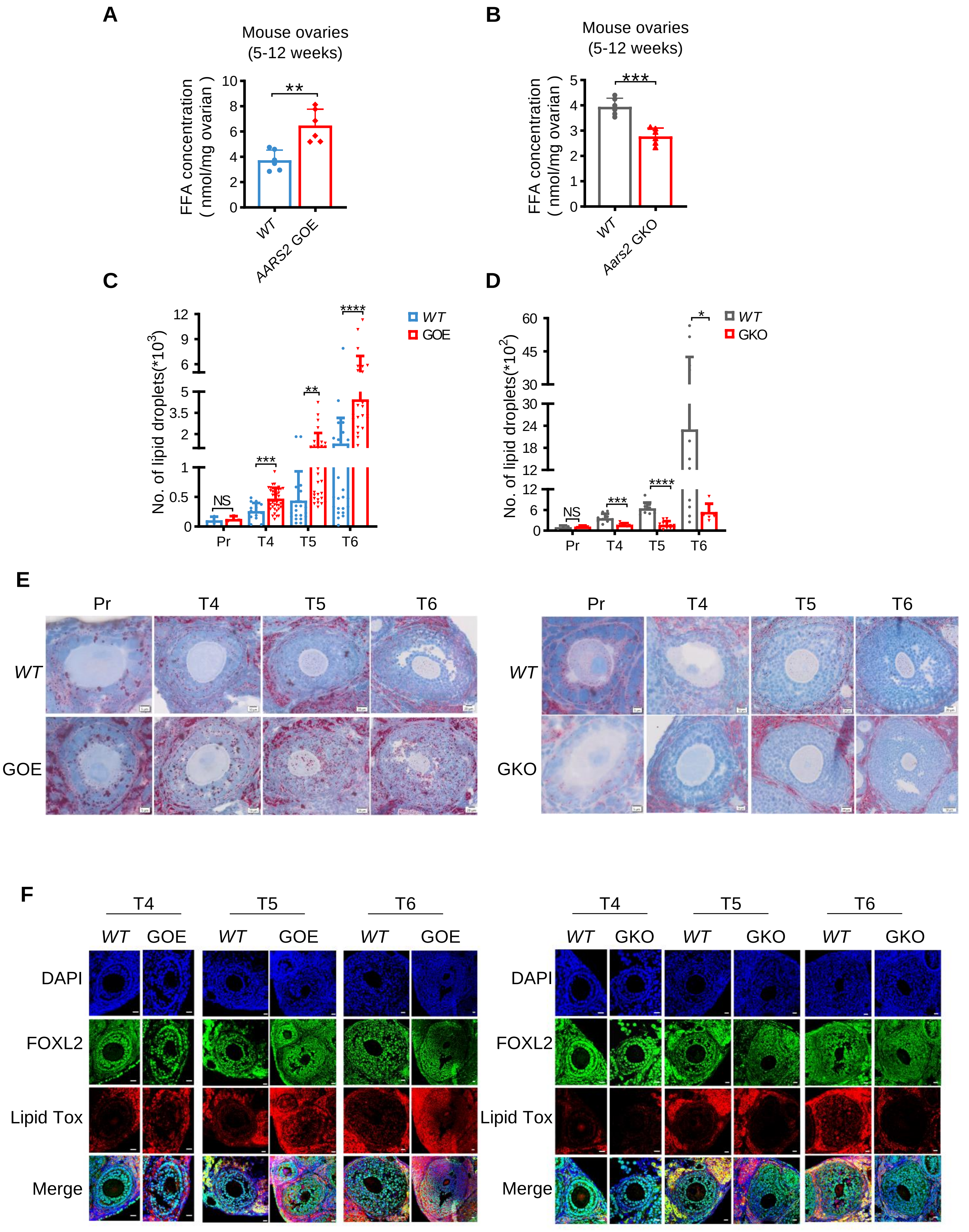
A
Mouse ovaries
(5-12 weeks)
FFA concentration
(nmol/mg ovarian)
**
WT
AARS2 GOE
B
Mouse ovaries
(5-12 weeks)
FFA concentration
(nmol/mg ovarian)
***
WT
Aars2 GKO
C
No. of lipid droplets(*10^3)
WT
GOE
NS
***
**
****
Pr
T4
T5
T6
D
No. of lipid droplets(*10^2)
WT
GKO
NS
***
****
*
Pr
T4
T5
T6
E
Pr
T4
T5
T6
WT
GOE
Pr
T4
T5
T6
WT
GKO
F
T4
T5
T6
WT
GOE
DAPI
FOXL2
Lipid Tox
Merge
T4
T5
T6
WT
GKO
DAPI
FOXL2
Lipid Tox
Merge

Supplementary Figure 5. AARS2 increases FFA and accumulates lipid droplets in mouse ovaries. Related to Figure 5.

(A-B) AARS2 induces FFA elevation in mouse ovaries. FFA concentration were detected in *WT*, *AARS2* GOE (**A**) and *Aars2* GKO (**B**) mouse ovaries (n=6).

(C-D) AARS2 accumulates lipid droplets in mouse GCs. Lipid droplets in GCs at different follicle stage of *WT*, *AARS2* GOE (**C**) and *Aars2* GKO (**D**) mice were quantified and presented as mean± SEM.

(E) AARS2 accumulates lipid droplets in mouse GCs. Representative images of *WT*, *AARS2* GOE, and *Aars2* GKO mouse ovarian sections, stained with oil red (scale bars, 5 μm (Pr), 10 μm (T4), 20 μm (T5 and T6)).

(F) AARS2 accumulates lipid droplets in mouse GCs. Representative images of *WT*, *AARS2* GOE, and *Aars2* GKO mouse ovarian sections, stained with GC marker FOXL2 and LipidTox (scale bars, 20 μm).

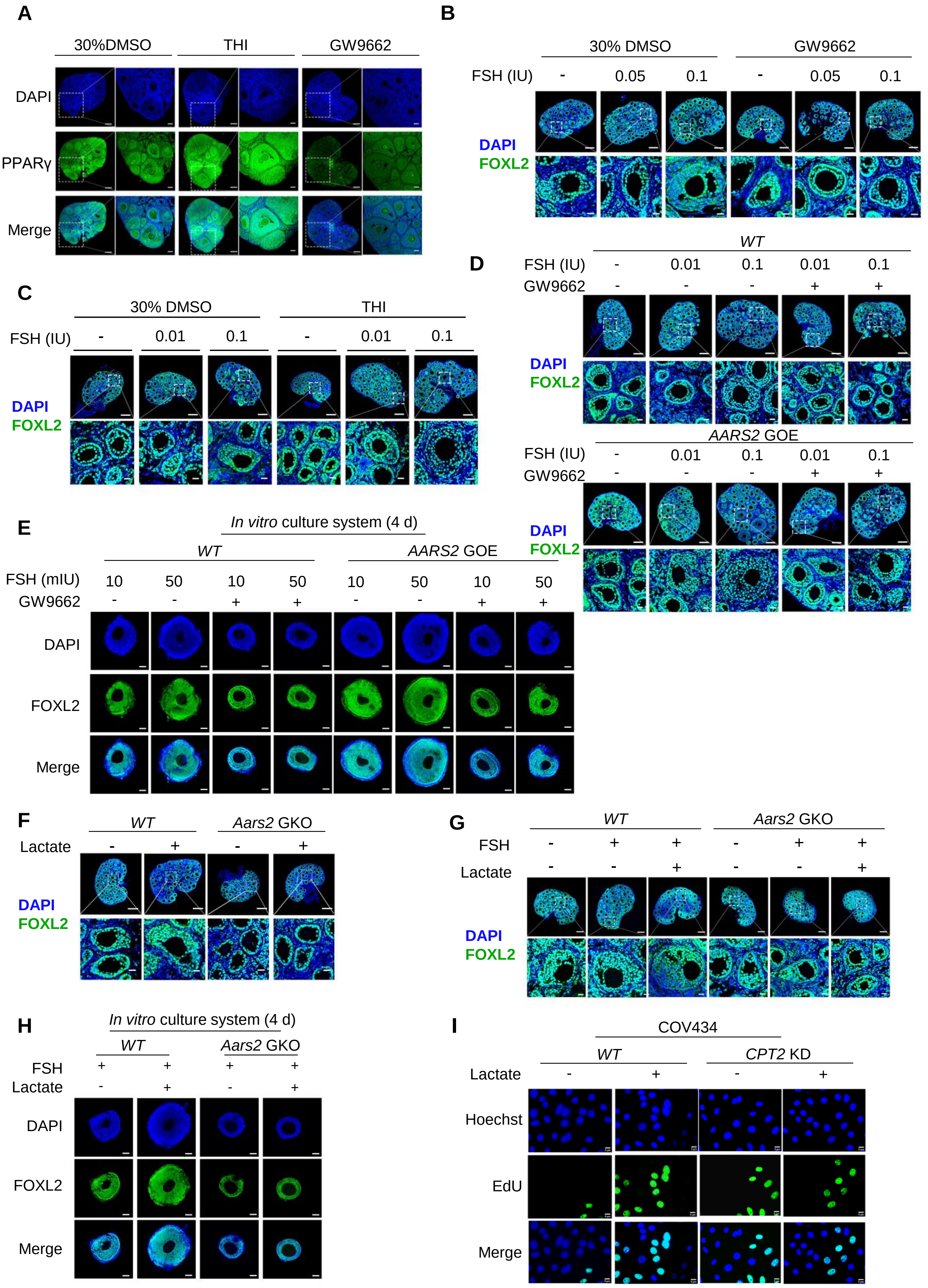
A
30%DMSO
THI
GW9662
DAPI
PPARγ
Merge
B
30% DMSO
GW9662
FSH (IU)
-
0.05
0.1
-
0.05
0.1
DAPI
FOXL2
C
30% DMSO
THI
FSH (IU)
-
0.01
0.1
-
0.01
0.1
DAPI
FOXL2
D
WT
FSH (IU)
-
0.01
0.1
0.01
0.1
GW9662
-
-
-
+
+
DAPI
FOXL2
AARS2 GOE
FSH (IU)
-
0.01
0.1
0.01
0.1
GW9662
-
-
-
+
+
DAPI
FOXL2
E
In vitro culture system (4 d)
WT
AARS2 GOE
FSH (mIU)
10
50
10
50
10
50
10
50
GW9662
-
-
+
+
-
-
+
+
DAPI
FOXL2
Merge
F
WT
Aars2 GKO
Lactate
-
+
-
+
DAPI
FOXL2
G
WT
Aars2 GKO
FSH
-
+
+
-
+
+
Lactate
-
-
+
-
-
+
DAPI
FOXL2
H
In vitro culture system (4 d)
WT
Aars2 GKO
FSH
+
+
+
+
Lactate
-
+
-
+
DAPI
FOXL2
Merge
I
COV434
WT
CPT2 KD
Lactate
-
+
-
+
Hoechst
EdU
Merge

Supplementary Figure 6. AARS2 and lactate sensitize follicles to FSH signaling. Related to Figure 5.

(A) THI increases and GW9662 decreases PPARγ signaling in mouse ovaries. Ovarian sections of wildtype mouse intraperitoneally injected with 30% DMSO, GW9662, or THI were stained with PPARγ, DAPI (scale bars, 200 μm (left panel), 50 μm (right panel)).

(B) FSH PPARγ-dependently increases FOXL2 signaling in mouse ovaries. Ovarian sections of wildtype mice intraperitoneally injected with 30% DMSO and GW9662 with 0, 0.05, and 0.1 IU FSH were stained with DAPI, FOXL2 (scale bars, 200 μm (top panel), 20 μm (bottom panel)).

(C) THI sensitizes ovaries' response to FSH signaling. Ovarian sections of wildtype mice intraperitoneally injected 30% DMSO and THI with 0, 0.01, and 0.1 IU FSH were stained with DAPI, FOXL2(scale bars, 200 μm (top panel), 20 μm (bottom panel)).

(D) GW9662 desensitizes AARS2's response to FSH signaling. Ovarian sections of *WT* and *AARS2* GOE female mice intraperitoneally injected 0, 0.01, 0.1IU FSH with or without GW9662 were stained with DAPI, FOXL2 (scale bars, 200 μm (top panel), 20 μm (bottom panel)).

(E) AARS2 PPARγ-dependently increases FOXL2 signaling in *in vitro* cultured follicles. Primary follicles of *WT* and *AARS2* GOE female mice cultured *in vitro* with 10 and 50 mIU FSH, DMSO or 10 μM GW9662 for 4 days were stained with FOXL2, DAPI (scale bars, 50 μm).

(F) Lactate AARS2-dependently increases FOXL2 signaling in mouse ovaries. Ovarian section of *WT* and *Aars2* GKO mice intraperitoneally injected PBS and 20 mg/kg lactate were stained with DAPI, FOXL2 (scale bars, 200 μm (top panel), 20 μm (bottom panel)).

(G) Lactate AARS2-dependently enhances FSH's action on mouse ovary. Ovarian section of *WT* and *Aars2* GKO mice intraperitoneally injected PBS, PBS and 0.01 IU FSH, 20 mg/kg lactate and 0.01 IU FSH were stained with DAPI, FOXL2 (scale bars, 200 μm (top panel), 20 μm (bottom panel)).

(H) Lactate AARS2-dependently increases FOXL2 signaling in *in vitro*-cultured follicles. Primary follicles of *WT* and *Aars2* GKO female mice cultured *in vitro* with 10 mIU FSH with or without 30 mM methyl-L-lactate for 4 days were stained with DAPI, FOXL2 (scale bars, 50 μm).

(I) Lactate increases EdU positive cells CPT2-dependently. *WT* and *CPT2* KD COV434 cells with PBS and methyl L-lactate treatment were stained with Hoechst, EdU (scale bars, 5 μm).

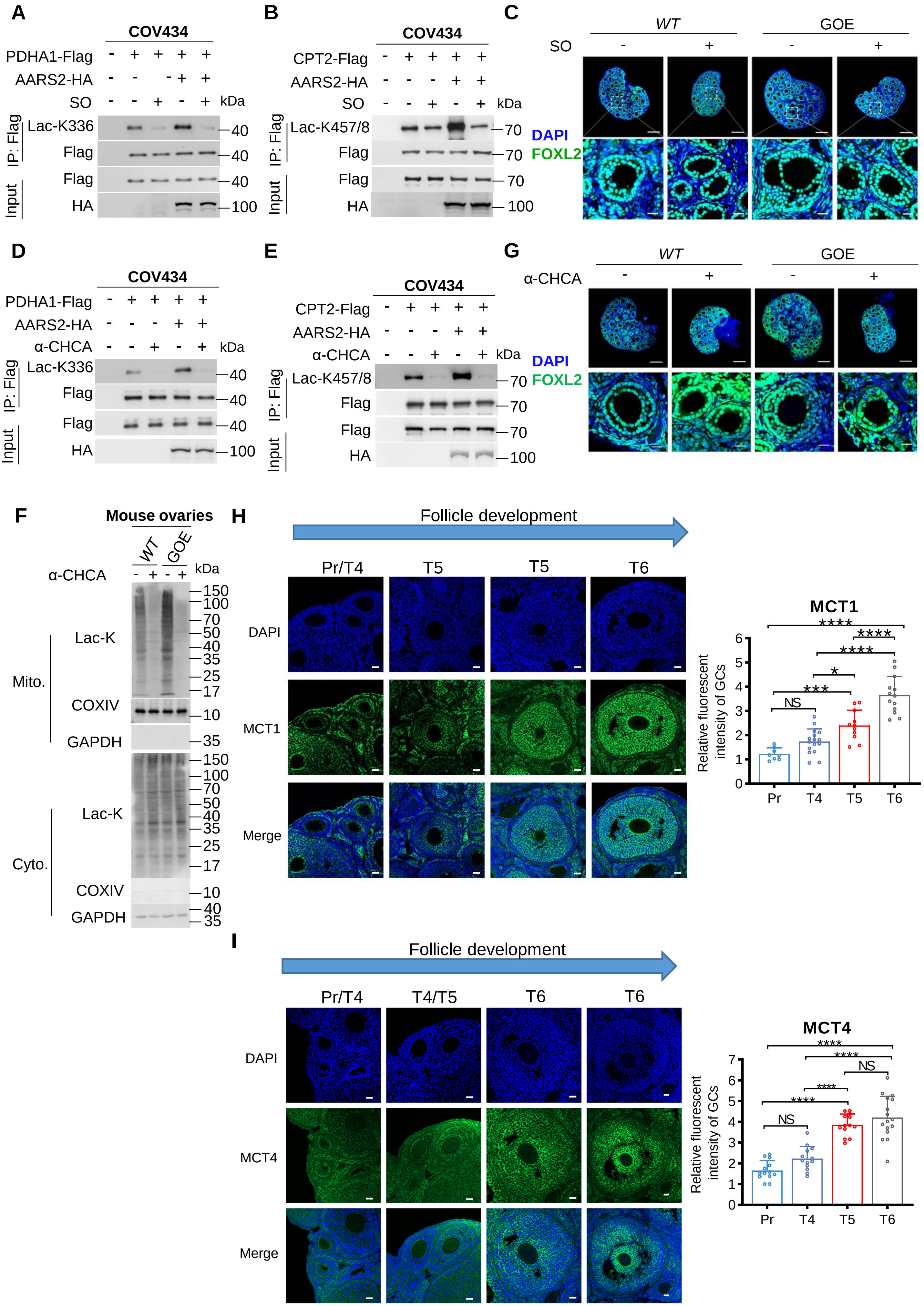
A
COV434
PDHA1-Flag
AARS2-HA
SO
kDa
IP: Flag
Lac-K336
Flag
Input
Flag
HA
B
COV434
CPT2-Flag
AARS2-HA
SO
kDa
Lac-K457/8
C
WT
GOE
DAPI
FOXL2
D
α-CHCA
E
G
F
Mouse ovaries
WT
GOE
Mito.
Lac-K
COXIV
GAPDH
Cyto.
H
Follicle development
Pr/T4
T5
T6
DAPI
MCT1
Merge
Relative fluorescent intensity of GCs
Pr
T4
NS
I
T4/T5
MCT4

Supplementary Figure 7. Inhibiting lactylation alleviates GCs proliferation. Related to Figure 6.

(A-B) SO rescues COV434 cells from high lactlylation induced by AARS2. PDHA1-Flag^{K336} (**A**)and CPT2-Flag$^{K457/8}$ (**B**) lactylation in COV434 cells transfected with empty vector and AARS2-HA with PBS or SO treatment were detected.

(C) SO inhibits FOXL2 signaling. Ovarian section of *WT* and *AARS2* GOE mice intraperitoneally injected PBS and 10 mg/kg SO were stained with DAPI, FOXL2 (scale bars, 200 μm (top panel), 20 μm (bottom panel)).

(D-E) α-CHCA rescues COV434 cells from high lactlylation induced by AARS2. PDHA1-Flag^{K336} (**D**)and CPT2-Flag$^{K457/8}$ (**E**) lactylation in COV434 cells transfected with empty vector and AARS2-HA with DMSO or α-CHCA treatment were detected.

(F) α-CHCA decreases pan Lac-K levels in ovarian mitochondrial. Pan lactylation in Ovarian plasma and mitochondrial of WT and GOE mice intraperitoneally injected DMSO and 10 mg/kg α-CHCA were detected.

(G) α-CHCA inhibits FOXL2 signaling. Ovarian section of *WT* and *AARS2* GOE mice intraperitoneally injected DMSO and 10 mg/kg α-CHCA were stained with DAPI, FOXL2 (scale bars, 200 μm (top panel), 20 μm (bottom panel)).

(H) GC MCT1 expression level increased during folliculogenesis. Ovarian section of *WT* mice were stained with DAPI, MCT1(scale bars, 20 μm) and relative MFI of MCT1 in Pr, T4, T5, T6 follicle GCs were analyzed (right panel).

(I) GC MCT4 expression level increased during folliculogenesis. Ovarian section of *WT* mice were stained with DAPI, MCT4(scale bars, 20 μm) and relative MFI of MCT4 in Pr, T4, T5, T6 follicle GCs were analyzed (right panel).

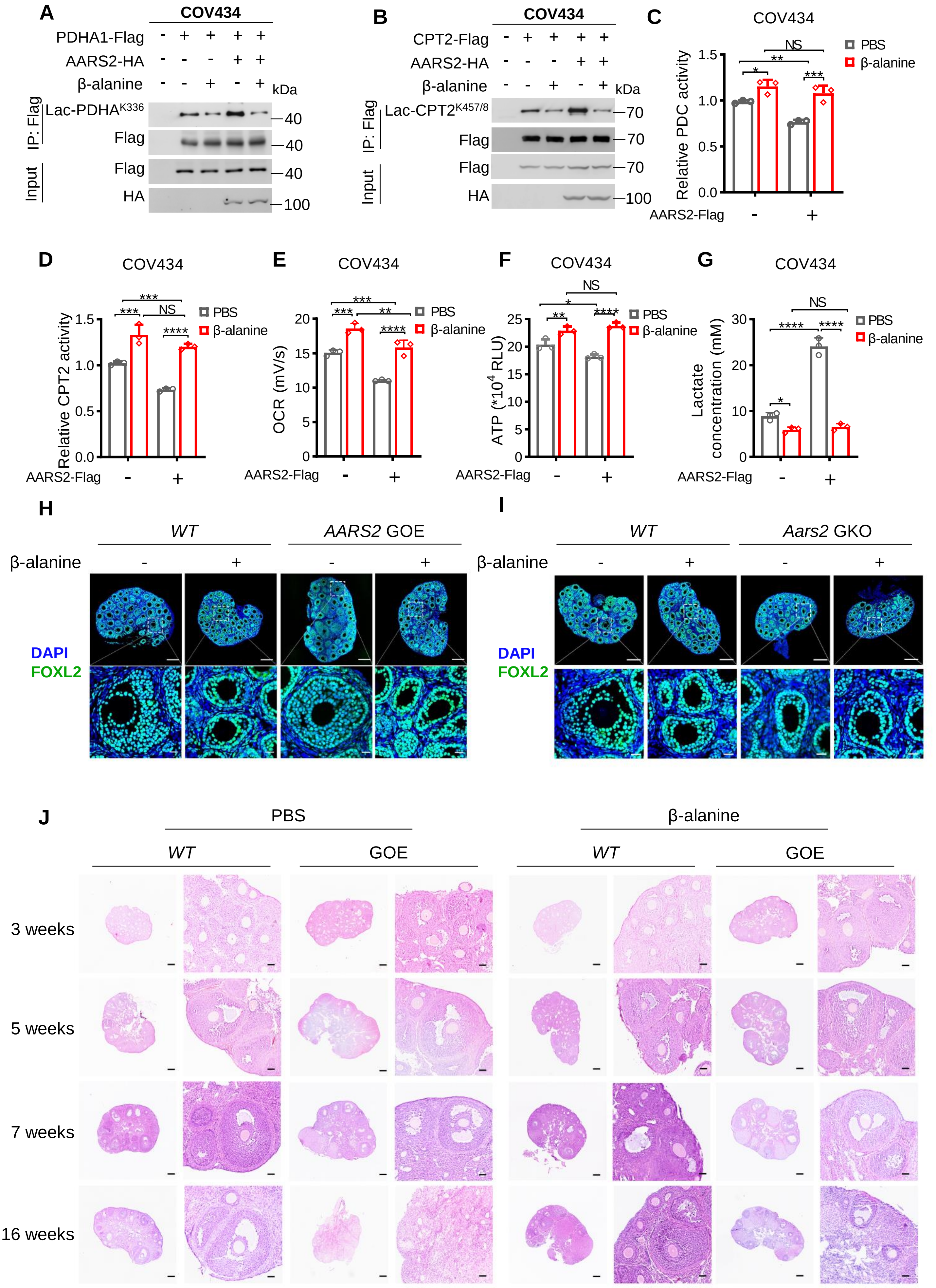
A
COV434
PDHA1-Flag
AARS2-HA
β-alanine
kDa
IP: Flag
Lac-PDHA K336
Flag
Input
Flag
HA
40
40
40
100
B
COV434
CPT2-Flag
AARS2-HA
β-alanine
kDa
IP: Flag
Lac-CPT2 K457/8
Flag
Input
Flag
HA
70
70
70
100
C
COV434
Relative PDC activity
PBS
β-alanine
NS
AARS2-Flag
D
COV434
Relative CPT2 activity
PBS
β-alanine
AARS2-Flag
E
COV434
OCR (mV/s)
PBS
β-alanine
AARS2-Flag
F
COV434
ATP (*10^4 RLU)
PBS
β-alanine
AARS2-Flag
G
COV434
Lactate concentration (mM)
PBS
β-alanine
AARS2-Flag
H
WT
AARS2 GOE
β-alanine
DAPI
FOXL2
I
WT
Aars2 GKO
β-alanine
DAPI
FOXL2
J
PBS
β-alanine
WT
GOE
WT
GOE
3 weeks
5 weeks
7 weeks
16 weeks

Supplementary Figure 8. β-alanine alleviates POI traits. Related to Figure 7.

(A-G) β-alanine rescues COV434 cells from high lactlylation induced by AARS2. PDHA^{K336} (**A**)and CPT2$^{K457/8}$ (**B**) lactylation, PDC (**C**), and CPT2 (**D**) enzyme activity, OCR(**E**), ATP content (**F**), and lactate content(**G**) in COV434 cells transfected with empty vector and AARS2-Flag/HA with PBS or β-alanine treatment were detected (n=3).

(H) β-alanine relieves FOXL2 signaling increases induced by AARS2. Ovarian section of *WT* and *AARS2* GOE mice intraperitoneally injected with PBS and β-alanine were stained with DAPI, FOXL2(scale bars, 200 µm (top panel), 20 µm (bottom panel)).

(I) β-alanine AARS2-dependently reduces FOXL2 signaling. Ovarian section of *WT* and *Aars2* GKO mice intraperitoneally injected with PBS and β-alanine were stained with DAPI, FOXL2(scale bars, 200 µm (top panel), 20 µm (bottom panel)).

(**J**) β-alanine increases ovarian reserve in *AARS2* GOE mice. Ovarian morphologies of PD 3-, 5-, 7-, 16-weeks were detected in *WT* and *AARS2* GOE mice with PBS and β-alanine intraperitoneal injection (scale bars, 200 µm (left panel), 50 µm (right panel)).

Table S1. Clinical Study Participants Information

characteristic	Healthy control (n = 61)	POI patients (n = 42)	*p* value
Age (years)	28.4800 ± 0.6357	29.7900 ± 1.0580	0.2631
AMH (ng/mL)	4.3650 ± 0.3684	0.0852 ± 0.0204	6.2403×10^{-16}
FSH (mIU/mL)	6.6370 ± 0.2824	82.1800 ± 4.2050	1.5548×10^{-40}
LH (mIU/mL)	7.1820 ± 0.5731	36.3100 ± 2.4130	2.8866×10^{-25}
FFA (mM)	0.3495 ± 0.0142	0.5664 ± 0.0289	5.0446×10^{-11}
Hypoxanthine (mM)	0.0231± 0.0009	0.0384± 0.0011	4.0906×10^{-19}
Formate (mM)	0.0451 ± 0.0017	0.0481± 0.0021	0.2771
Citrate (mM)	0.1748 ± 0.0044	0.1464 ± 0.0032	4.6555×10^{-6}
Creatine (mM)	0.1257 ± 0.0026	0.1522 ± 0.0037	2.0974×10^{-8}
Tryptophan (mM)	0.0811 ± 0.0016	0.0926 ± 0.0020	1.9319×10^{-5}
Inosine (mM)	0.0097± 0.0004	0.0130± 0.0006	1.9529×10^{-5}
Glycerophocholine/ Phosphorylcholine (mM)	0.3216 ± 0.0090	0.3581 ± 0.0119	0.0141
Choline (mM)	0.0477 ± 0.0019	0.0717± 0.0021	4.7274×10^{-13}
Asparagine (mM)	0.4304 ± 0.0105	0.5307 ± 0.0206	7.5382×10^{-6}
Pyruvate (mM)	0.0550 ± 0.0028	0.0959 ± 0.0038	2.0293×10^{-14}
Dimethylamine (mM)	0.0371 ± 0.0017	0.0467± 0.0019	1.8204×10^{-5}
Histidine (mM)	0.0963± 0.0014	0.0791 ± 0.0012	2.4154×10^{-14}

Phenylalanine (mM)	0.1355 ± 0.0023	0.0861 ± 0.0022	1.2584×10^{-27}
Tyrosine (mM)	0.0825 ± 0.0020	0.1074 ± 0.0027	1.2865×10^{-11}
Glucose (mM)	5.1590± 0.0622	4.7710± 0.2911	0.1274
Lactate (mM)	3.2800± 0.1018	6.2630± 0.1478	8.9884×10^{-32}
Glycine (mM)	0.6148 ± 0.0928	0.6366 ± 0.0235	0.8479
Glutamine (mM)	1.0800± 0.0197	0.9501 ± 0.0154	5.1985×10^{-6}
Acetate (mM)	0.1365 ± 0.0031	0.1638 ± 0.0034	6.5642×10^{-8}
Alanine (mM)	0.6980 ± 0.0159	0.5252 ± 0.0114	1.4194×10^{-12}
Valine (mM)	0.4116 ± 0.0089	0.3540 ± 0.0092	2.9531×10^{-5}
Isoleucine (mM)	0.2111 ± 0.0056	0.1752 ± 0.0053	2.3621×10^{-5}
Leucine (mM)	0.1966 ± 0.0042	0.1570 ± 0.0041	2.6861×10^{-9}
3-Hydroxybutytrate (mM)	0.3789 ± 0.0163	0.4228 ± 0.0248	0.1264

AMH: Anti-Müllerian Hormone, FSH: Follicle Stimulating Hormone, LH: Luteinizing Hormone, FFA: Free Fatty Acids,

Values are expressed as mean + SEM. The statistical significance of differences (*p* value) was determined by unpaired two-tailed Student’s t-test.

Table S1, Clinical Study Participants Information

The age, hormonal and biochemical information of POI patients (n=42) and matched control women (n=61) are presented.

**Table S2, Oligonucleotide information**	
Name	Source
CRISPR/Cas9-mediated AARS2 gene editing primer-1-F: CACCGCTAGCCGTGTGCTTTCCTT	This Study
CRISPR/Cas9-mediated AARS2 gene editing primer-1-R: AAACAAGGAAAGCACACGGCTAGC	This Study
CRISPR/Cas9-mediated AARS2 gene editing primer-2-F: CACCGACACGGCTAGCAGGCACCCT	This Study
CRISPR/Cas9-mediated AARS2 gene editing primer-2-R: AAACAGGGTGCCTGCTAGCCGTGTC	This Study
sh*PDHA1*#1 sequence: CCGGGCCAATCAGTGGATCAAGTTTCTCGAGAAACTTGATCCACT GATTGGCTTTTTG	This Study
sh*PDHA1*#2 sequence: CCGGCGAATGGAGTTGAAAGCAGATCTCGAGATCTGCTTTCAACT CCATTCGTTTTTG	This Study
sh*CPT2*#1 sequence: CCGGCTCCGTTGTTCTGAACTTTAACTCGAGTTAAAGTTCAGAACA ACGGAGTTTTTG	This Study
sh*CPT2*#2 sequence: CCGGTTGTCCACTTGTCCCACAATACTCGAGTATTGTGGGACAAG TGGACAATTTTTG	This Study
shScramble sequence: CCGGCCTAAGGTTAAGTCGCCCTCGCTCGAGCGAGGGCGACTTA ACCTTAGGTTTTTG	This Study
AARS2 cloning primer-F: AACGGGCCCTCTAGACTCGAGATGGCAGCGTCAGTGGCA	This Study
AARS2 cloning primer-R: TAGTCCAGTGTGGTGGAATTCGAGCTGGCTGAGGGCATAGG	This Study
AARS2 R199C site-directed mutagenesis primer-F: CTGCTAGCTGTGTGCTTTCCTTTGGACCACAA	This Study
AARS2 R199C site-directed mutagenesis primer-R: AAGCACACAGCTAGCAGGCACCCCTAAGCTCA	This Study
AARS2 F50C site-directed mutagenesis primer-F: CTGCTAGCTGTGTGCTTTCCTTTGGACCACAA	This Study
AARS2 F50C site-directed mutagenesis primer-R: GTCCCGACAGAAGTTCAGAAAGGCGGCCCTCA	This Study
AARS2 A77V site-directed mutagenesis primer-F: TGTCAATGTGGGCATGAACCAGTTCAAGCCAA	This Study
AARS2 A77V site-directed mutagenesis primer-R: TCATGCCCACATTGACAAAAAGCAAACTGGGG	This Study
AARS2 N104Y site-directed mutagenesis primer-F: TGGCCTACAGCCAGAAATGTGTGAGAGCTGGA	This Study

AARS2 N104Y site-directed mutagenesis primer-R: TTTCTGGCTGTAGGCCACACGTCGGAAGCCTG	This Study
AARS2 T382K site-directed mutagenesis primer-F: TGGTGGAGAAACTGGGAGATGCTTATCCAGAACT	This Study
AARS2 T382K site-directed mutagenesis primer-R: TCCCAGTTTCTCCACCACTACAGGTACCAGGC	This Study
CPT2 cloning primer-F: CGCTCTAGCCCGGGCGGATCCCCACCATGGTGCCCCGCCTGCTG	This Study
CPT2 cloning primer-R: TCTGTCGACGATATCGAATTCACTTTTGATGGATTTGCCTTCT	This Study
PDHA1 cloning primer-F AACGGGCCCTCTAGACTCGAGATGAGGAAGATGCTCGCCG	This Study
PDHA1 cloning primer-R TAGTCCAGTGTGGTGGAATTCACTTTTGATGGATTTGCCTTCT	This Study
CPT2 K457/8R site-directed mutagenesis primer-F: TTCCTGAGGAGGCAAAAGCTGAGCCCTGACGC	This Study
CPT2 K457/8R site-directed mutagenesis primer-R: CTTTTGCCTCCTCAGGAATTCTTTGCCTCCTCTCT	This Study
AARS2-OE (genotyping)-Primer1-F: TCAGATTCTTTTATAGGGGACACA	This Study
AARS2-OE (genotyping)-Primer1-R: TAAAGGCCACTCAATGCTCACTAA	This Study
AARS2-OE (genotyping)-Primer2-F: TGCCCTCAGTATAGCCCAAACC	This Study
AARS2-OE (genotyping)-Primer2-R: GCAGCCAAGGAAAGGACGATGATT	This Study
$Amhr2^{cre}/Zp3^{cre}$ (genotyping)-Primer-F: TGCCACGACCAAGTGACAGCAATG	This Study
$Amhr2^{cre}/Zp3^{cre}$ (genotyping)-Primer-R: *AGAGACGGAAATCCATCGCTCG*	This Study
Aars2-KO (genotyping)-Primer1-F: AAGCAACAGGAGAAGAGGTGTTGG	This Study
Aars2-KO (genotyping)-Primer1-R: TAACCATCTCAGCAGCCCAGCAT	This Study
Aars2-KO (genotyping)-Primer2-F: TTTCTATTTGAGCCTCTGTACCAAGC	This Study
Aars2-KO (genotyping)-Primer2-R: TCTGAGGCGGAAAGAACCAG	This Study

Table S2, Oligonucleotide information

The sequences and source of the oligonucleotides used in this study are listed.

Table S3 Plasmids used for recombinant DNA		
Name	Source	GenBank Name
pcDNA3.1b-AARS2-Flag/HA	This Study	NM_020745.4
pcDNA3.1b-PDHA1-Flag	This Study	NM_000284.4
pET28b-His-AARS2	This Study	NM_020745.4
PCMV-CPT2-Flag	This Study	NM_000098.3
PCDH-AARS2-Flag/HA	This Study	NM_020745.4

Table S3, Plasmid information

The name, source, and GenBank number of the plasmid used in this study are listed.
