# Supplementary material for: AARS2-catalyzed lactylation induces follicle development and premature ovarian insufficiency
Source: Cell Death Discov. 2025 Apr 29;11:209. doi: 10.1038/s41420-025-02501-0 (PMC12041370; doi:10.1038/s41420-025-02501-0)

Source Data Figure 3A

FIG. 3A kDa

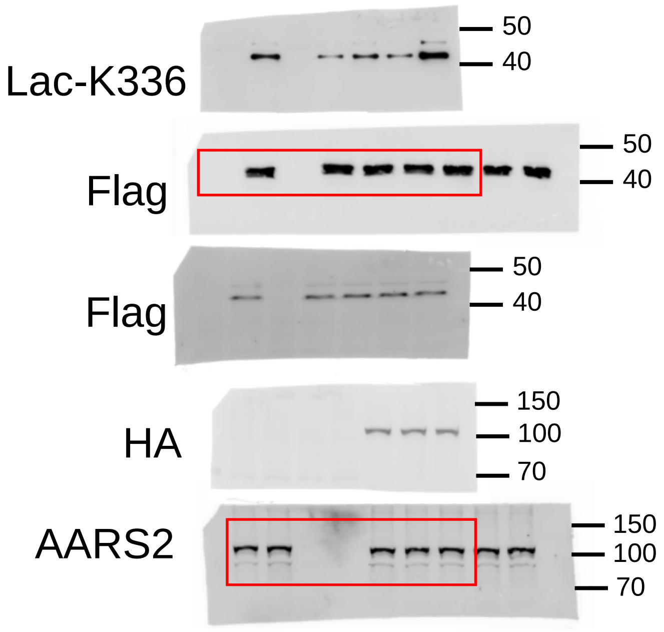

Source Data Figure 3B

Fig. 3B kDa

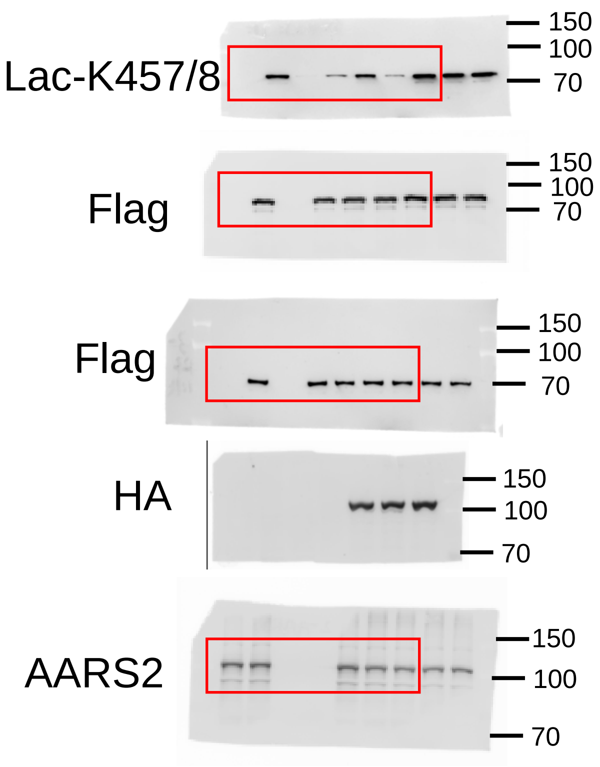

Source Data Figure 3C

Fig. 3C

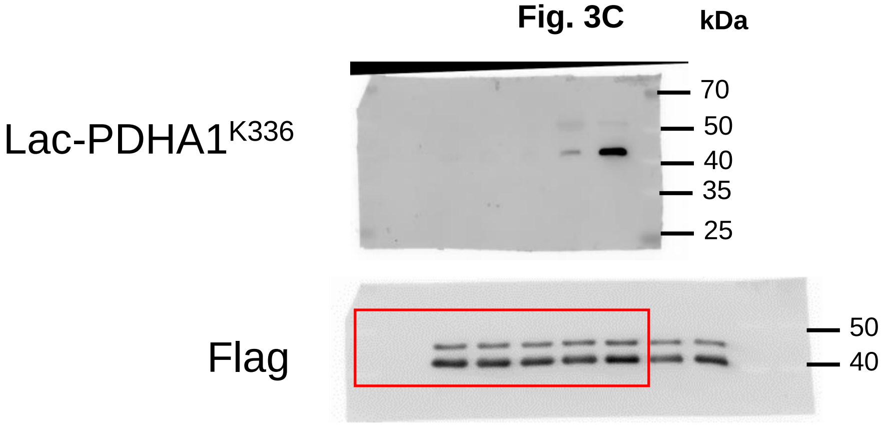

Source Data Figure 3D

Fig. 3D

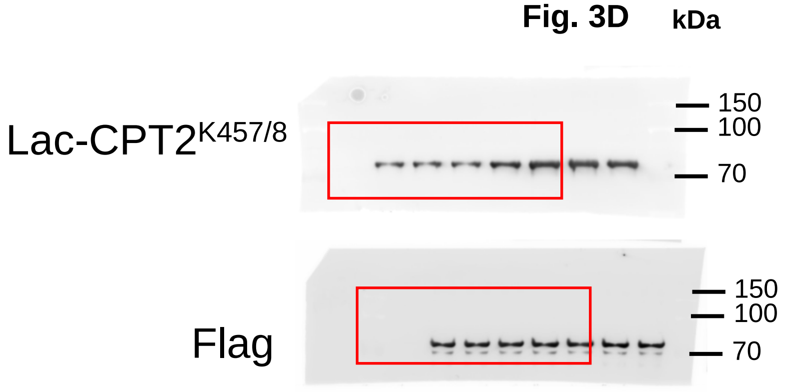

Source Data Figure 3E

Fig. 3E kDa

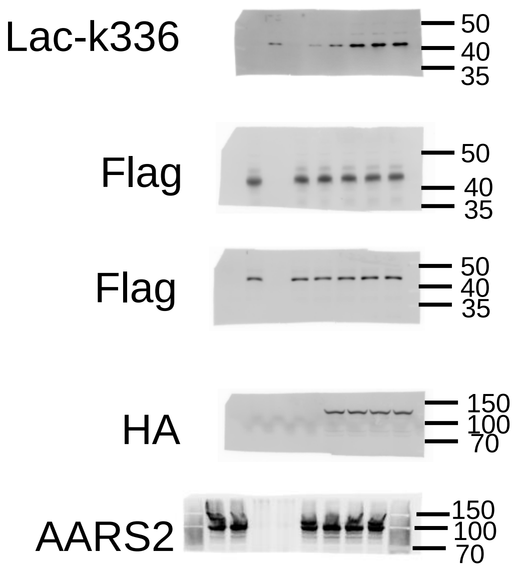

Source Data Figure 3F

Fig. 3F kDa

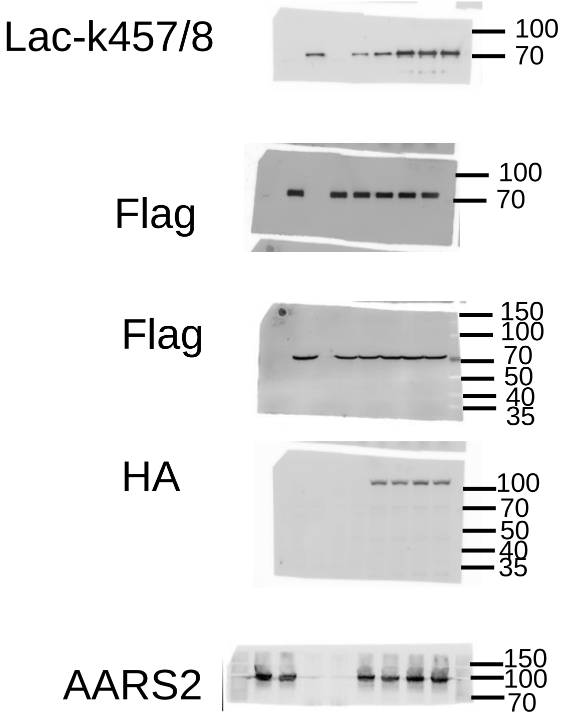

Source Data Figure 3I

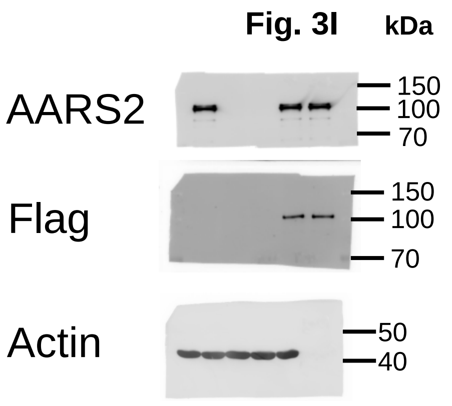

Source Data Figure 3J

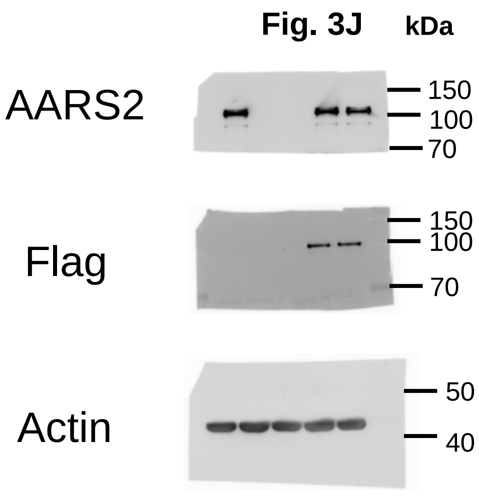

Source Data Figure 4M

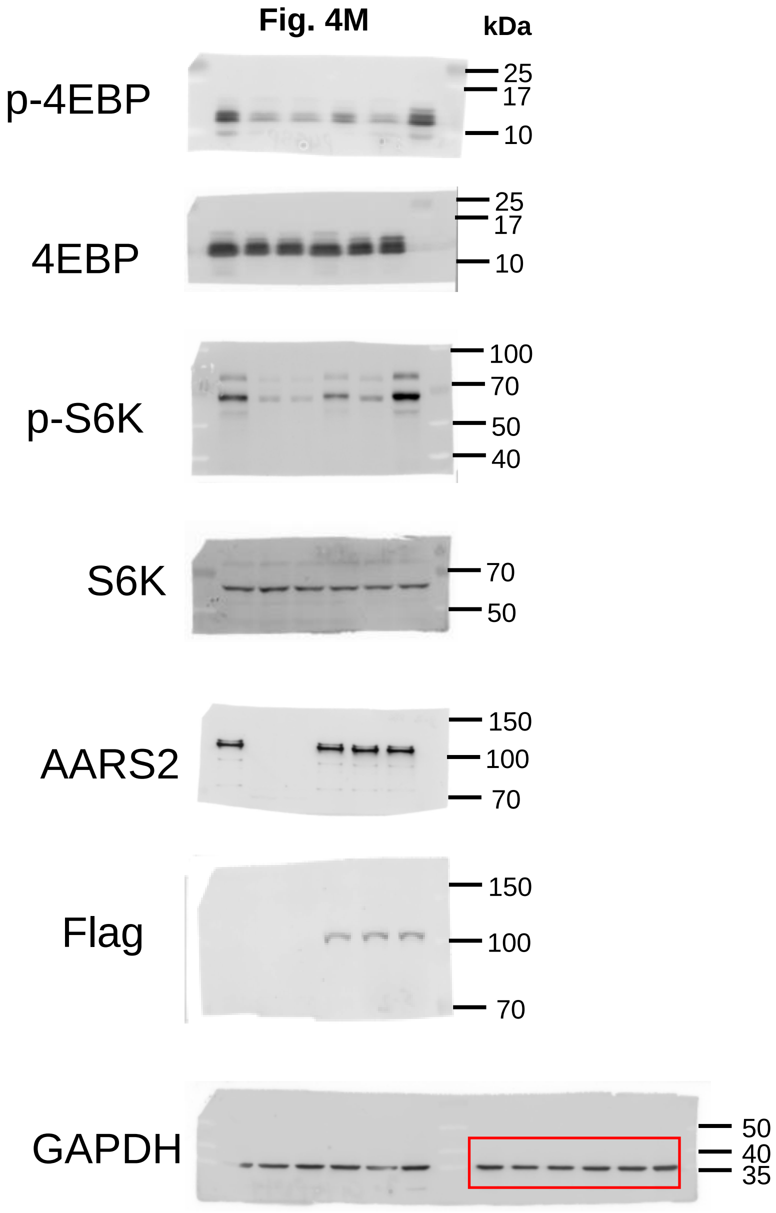

Source Data Figure 4N

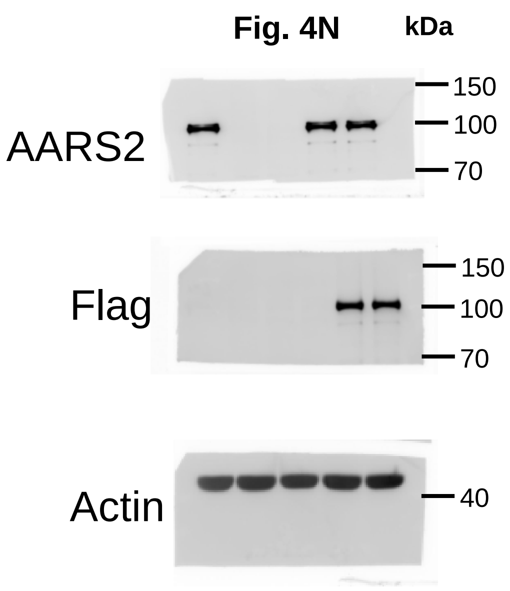

Source Data Figure 4O

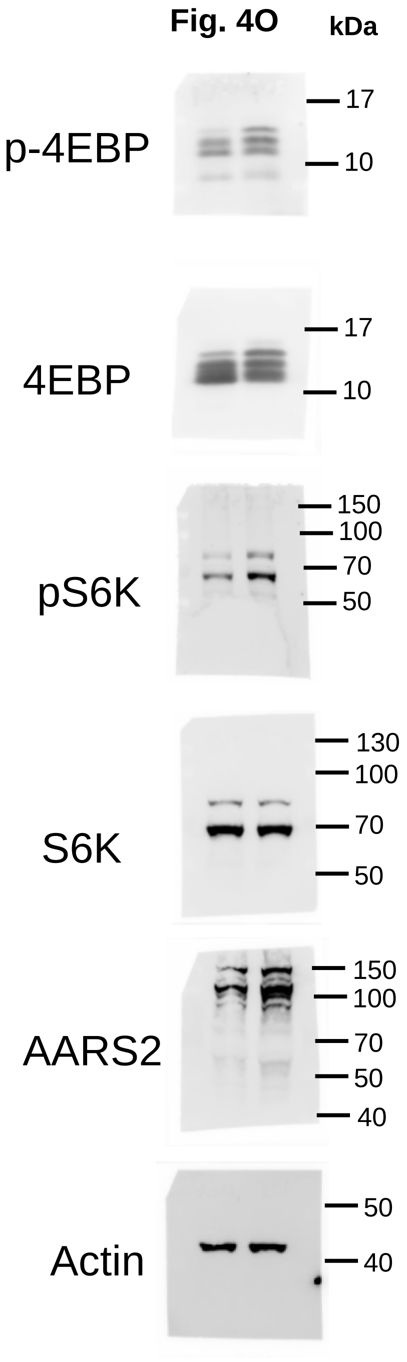

Source Data Figure 5A

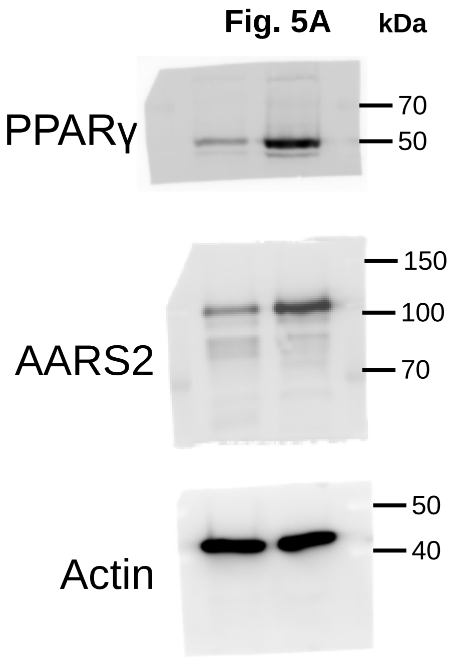

Source Data Figure 5B

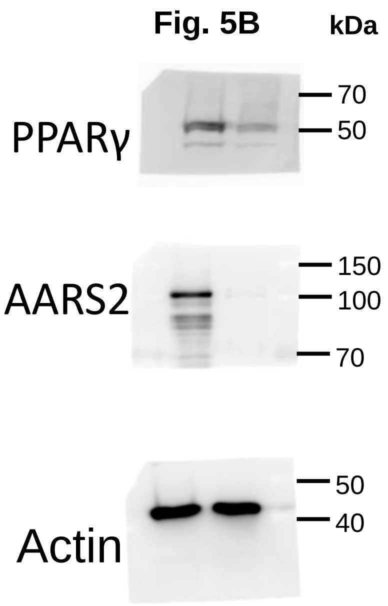

Source Data Figure 5C

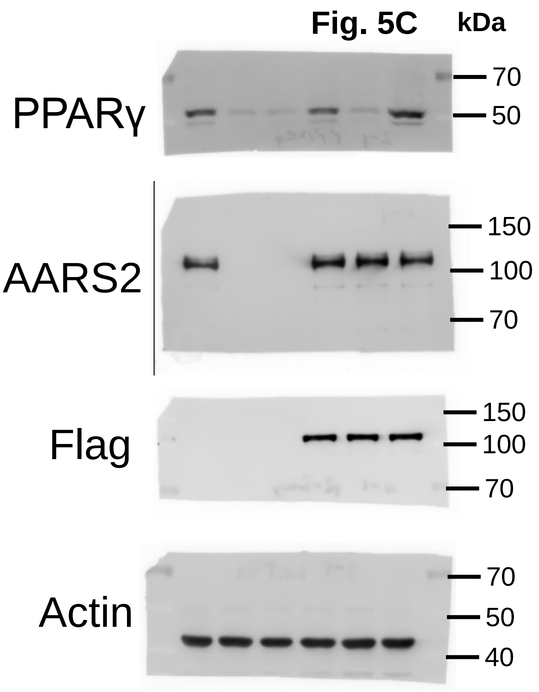

Source Data Figure 5D

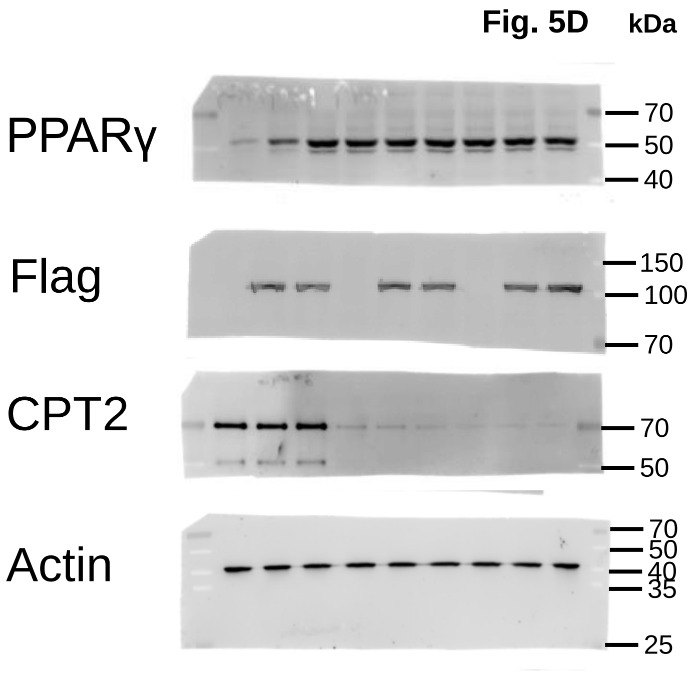

Source Data Figure 5E

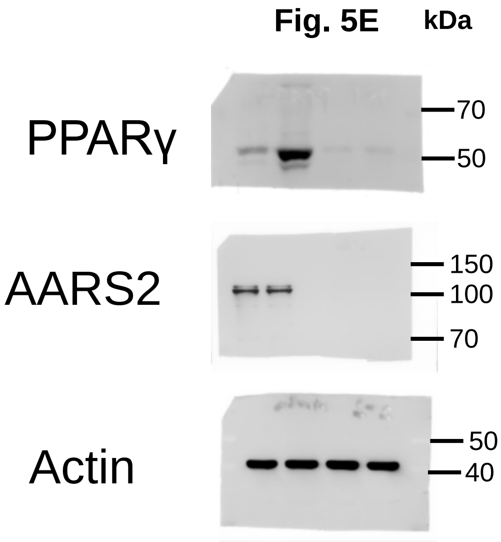

Source Data Figure 5G

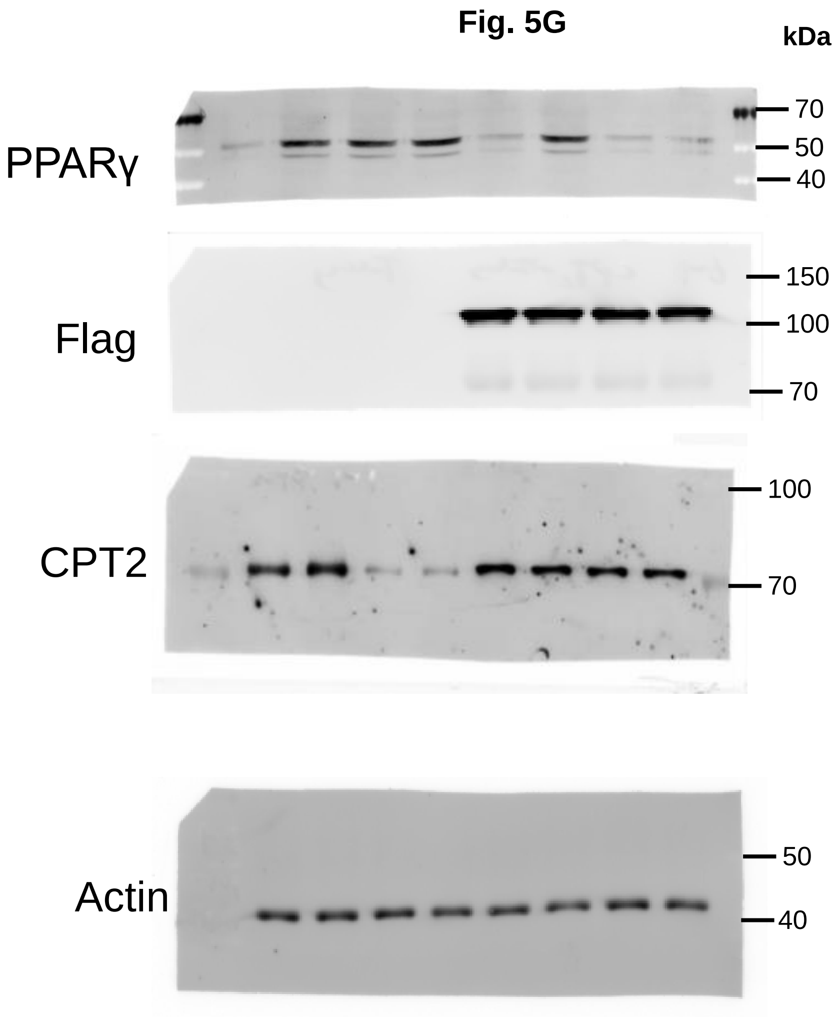

Source Data Figure 6D

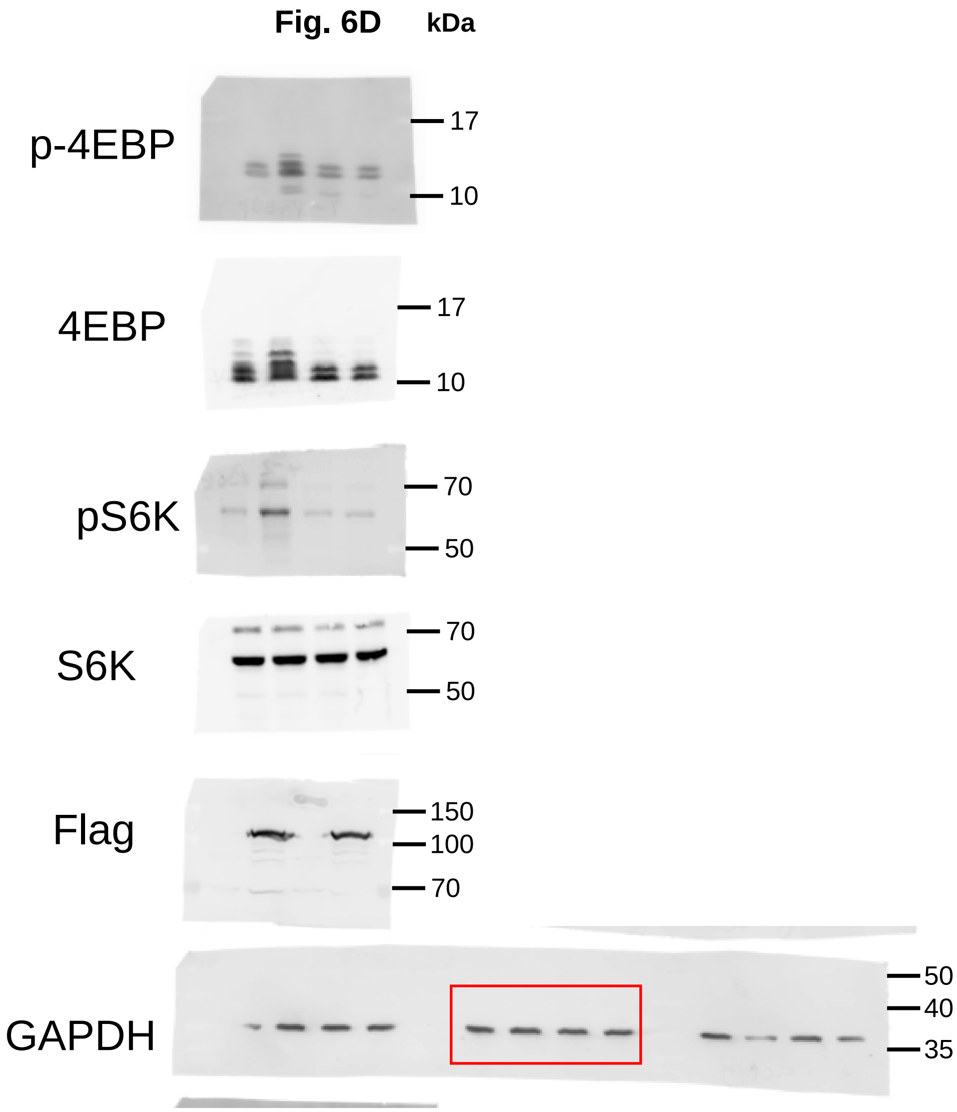

Source Data Figure 6F

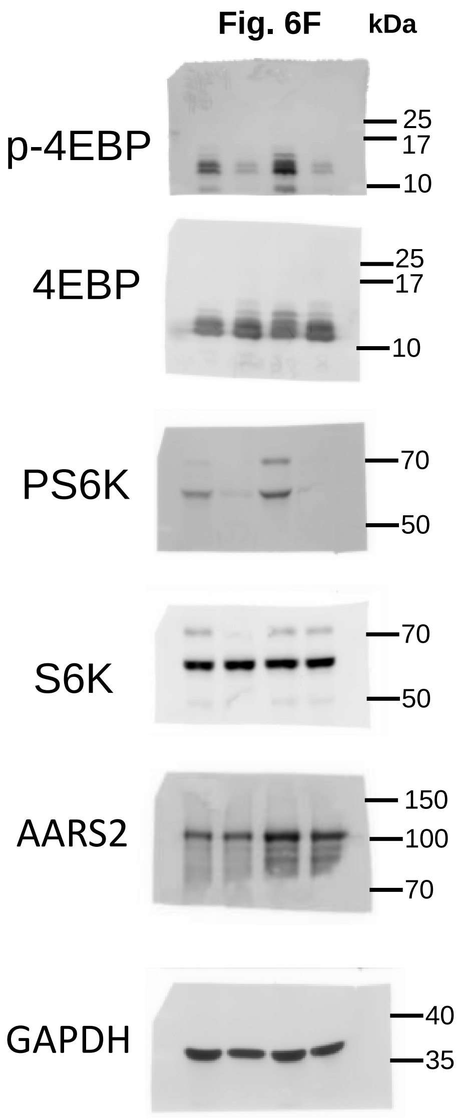

Source Data Figure 6J

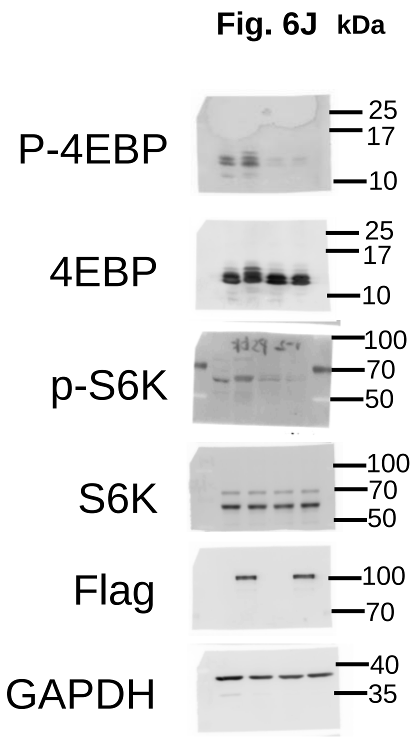

Source Data Figure 6L

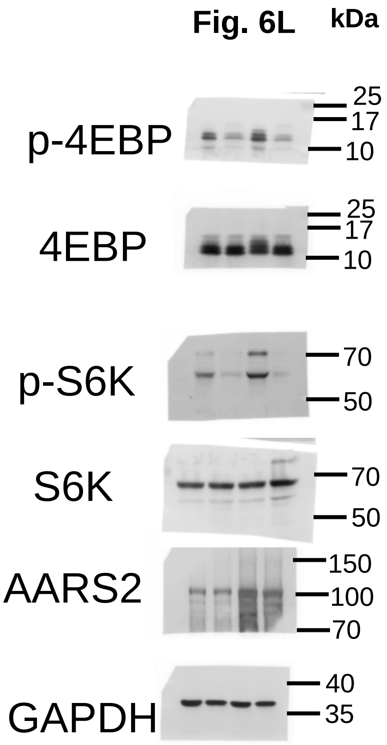

Source Data Figure 6N

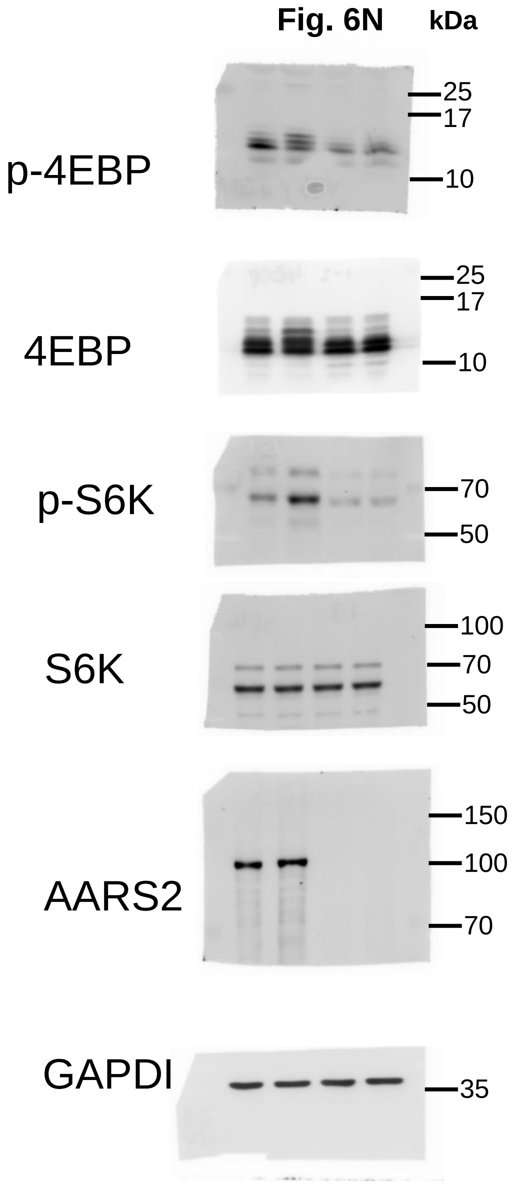

Source Data Figure 6O

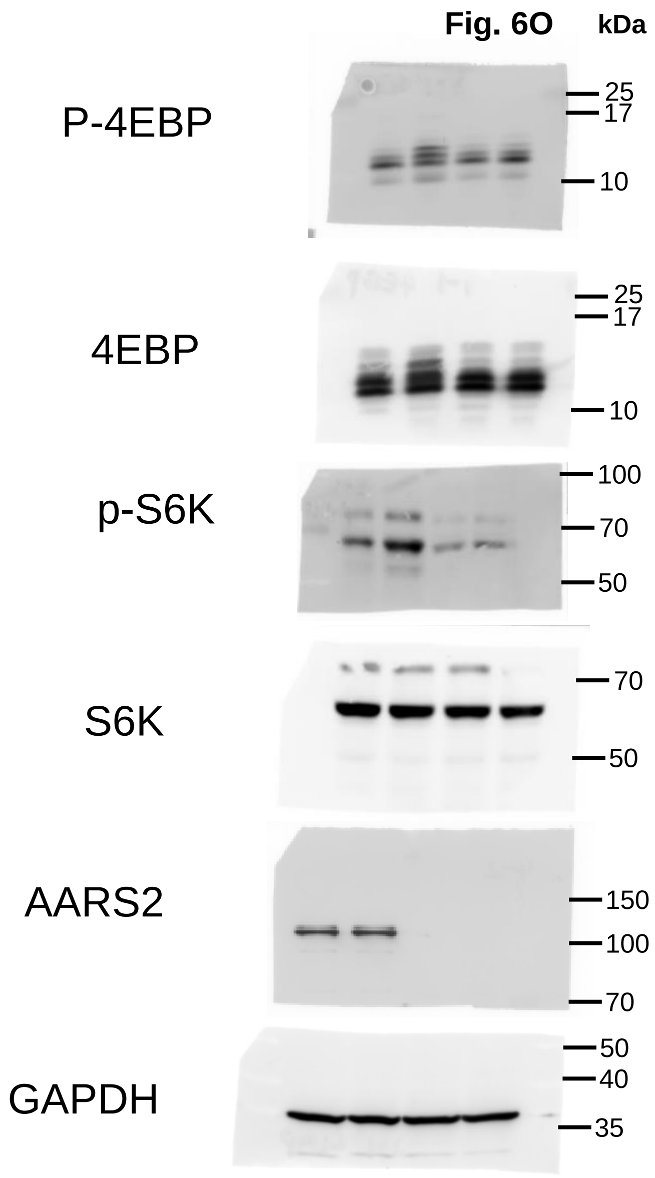

Source Data Figure 7A

Fig. 7A

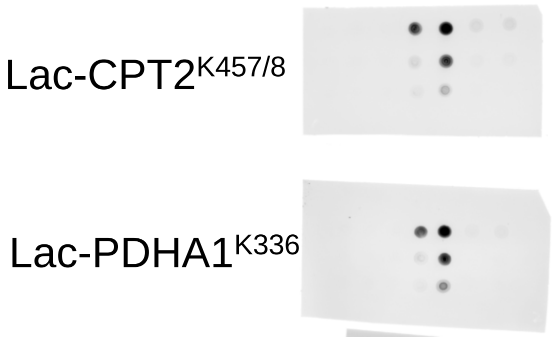

Source Data Figure 7B

Fig. 7B

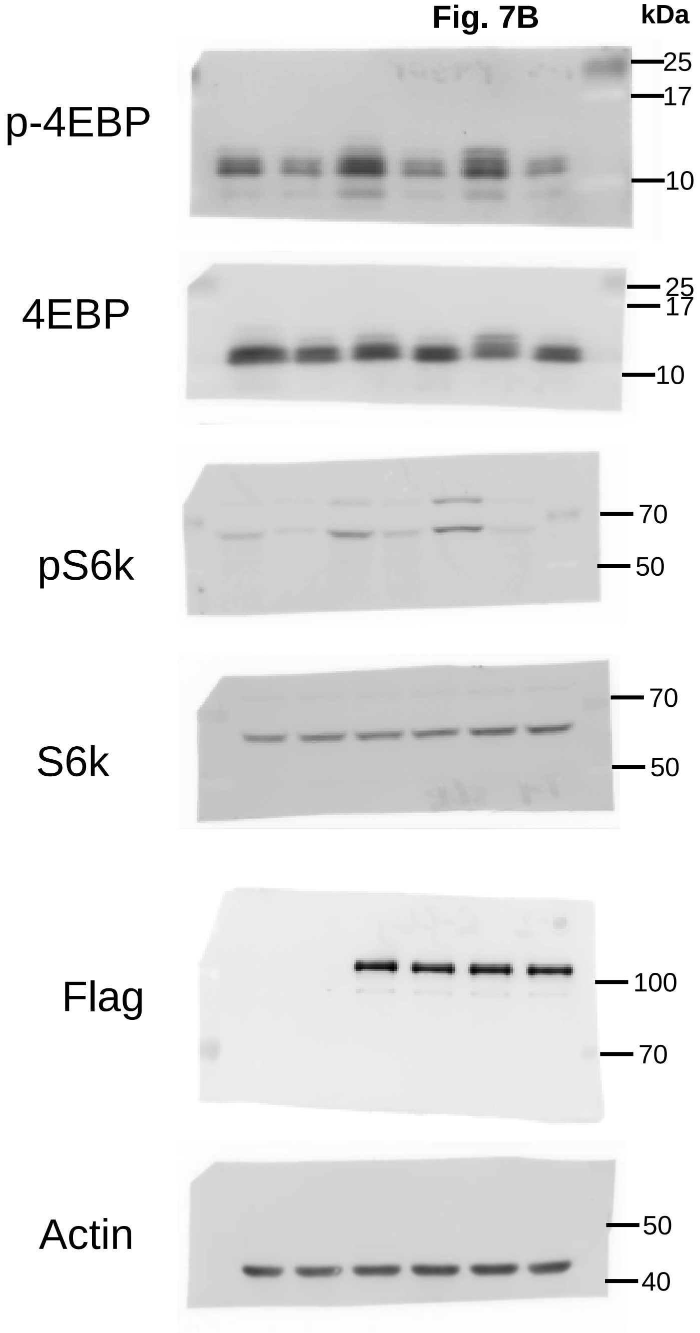

Source Data Figure 7D

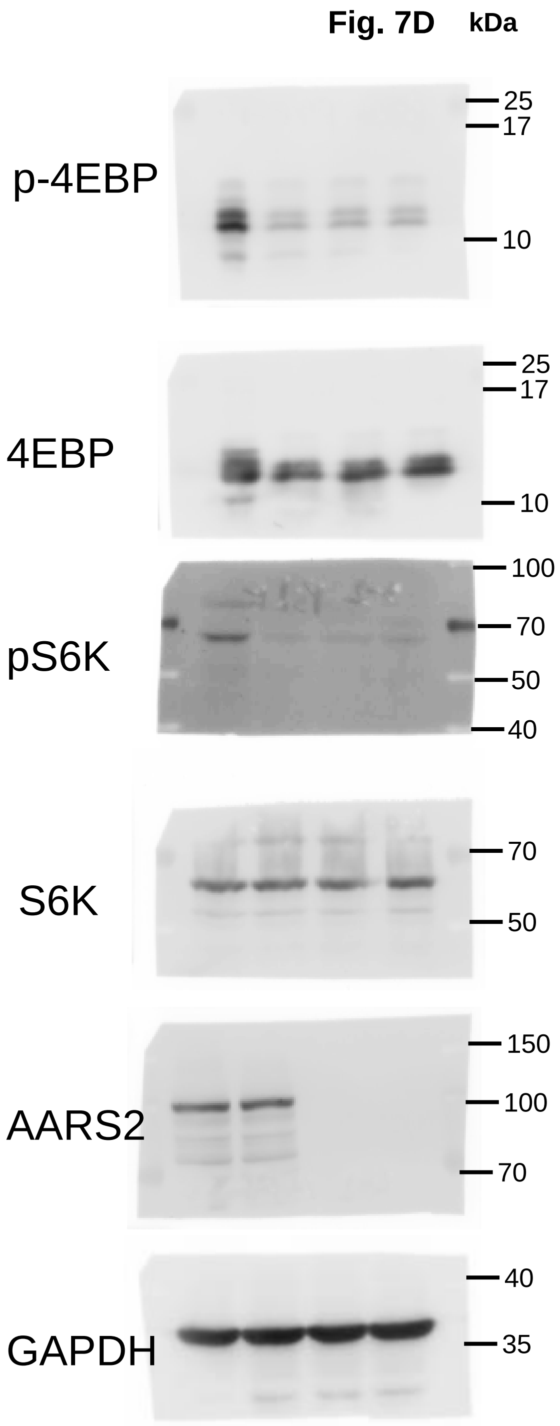

Source Data Figure 7F

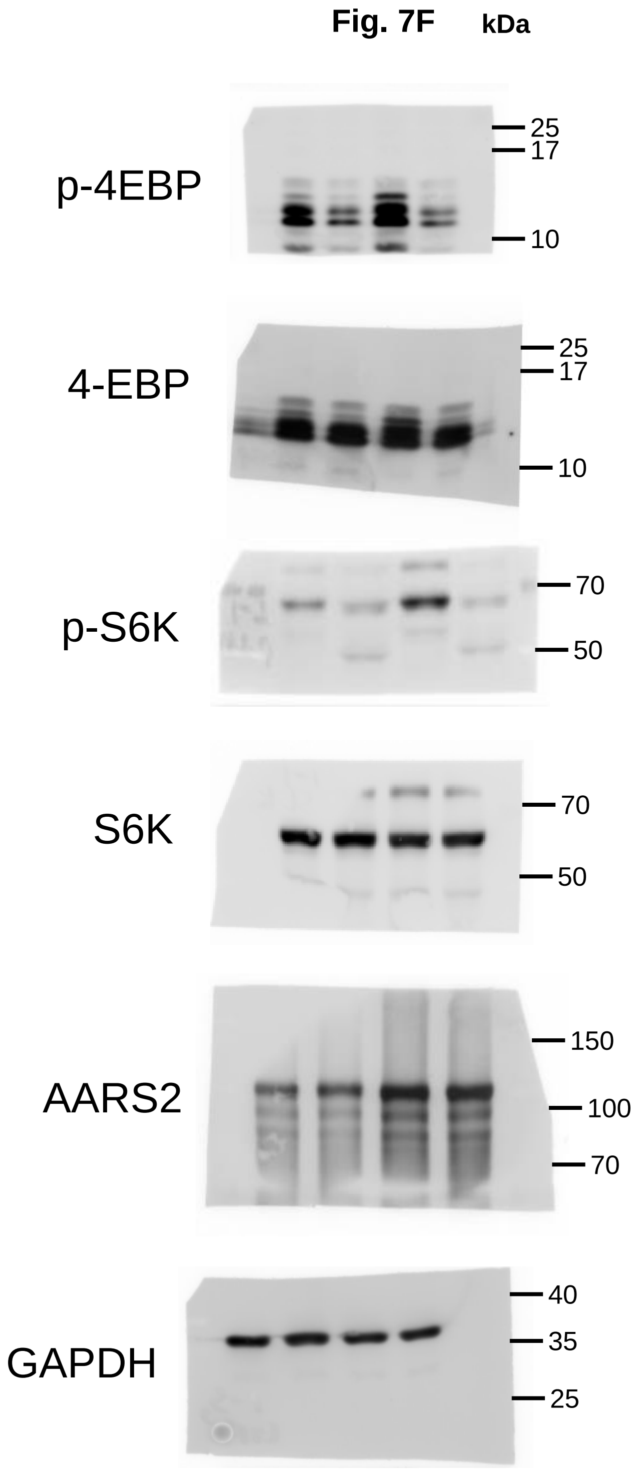

Source Data Figure 7I

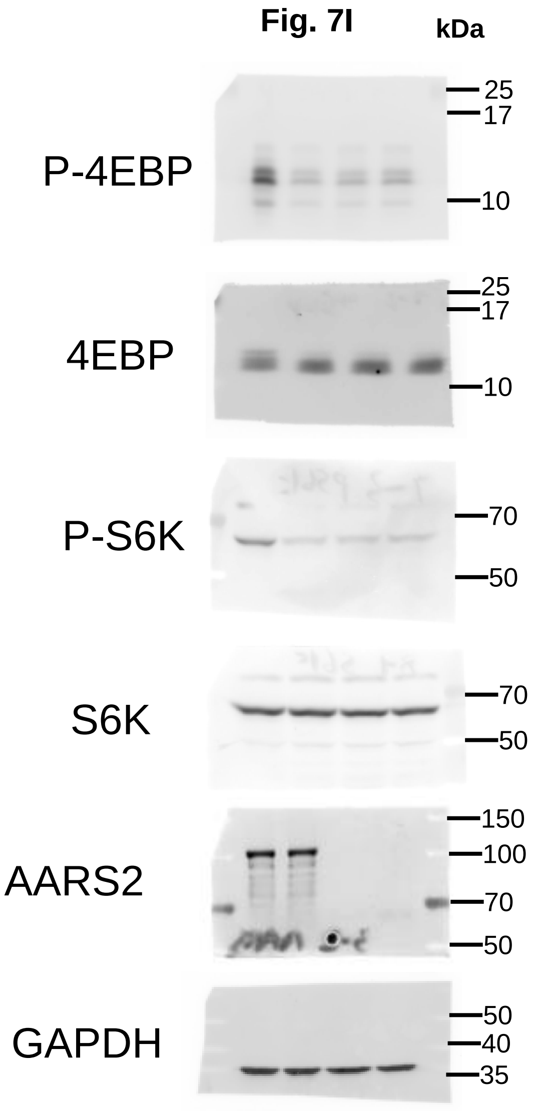

Source Data Figure S3A

Fig. S3A

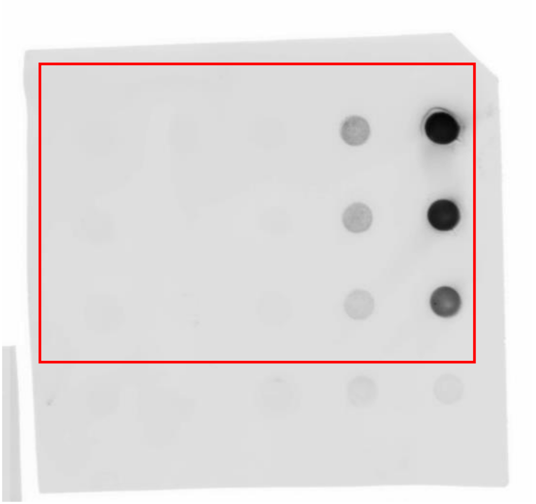

Source Data Figure S3B

Fig. S3B

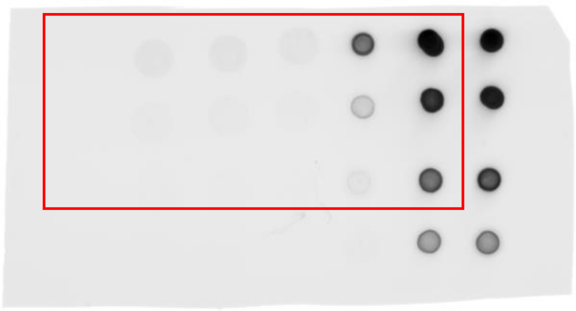

Source Data Figure S3C

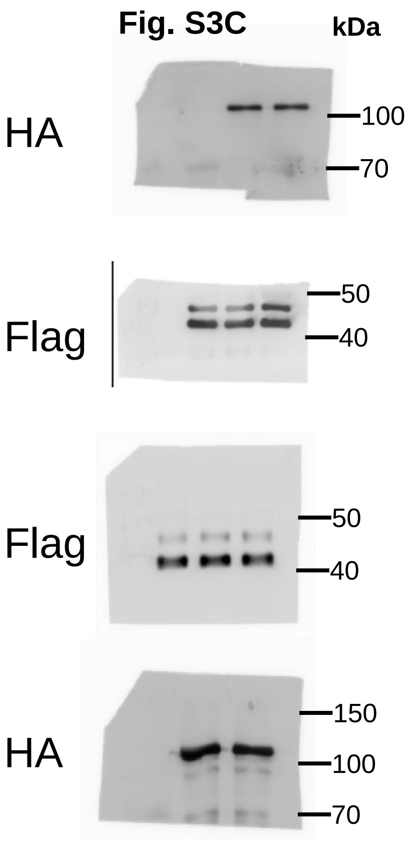

Source Data Figure S3D

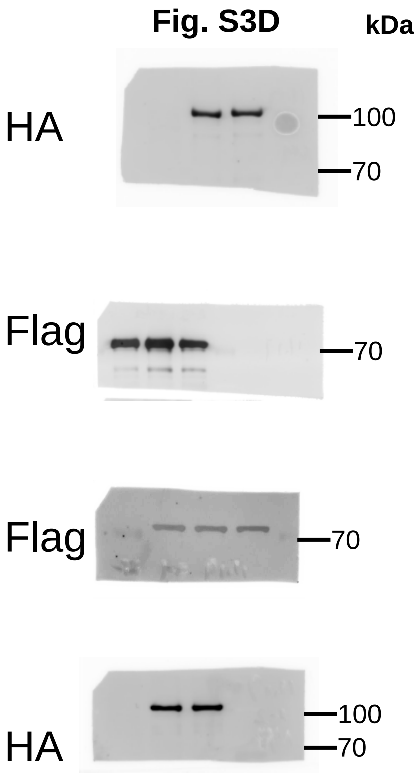

Source Data Figure S3E

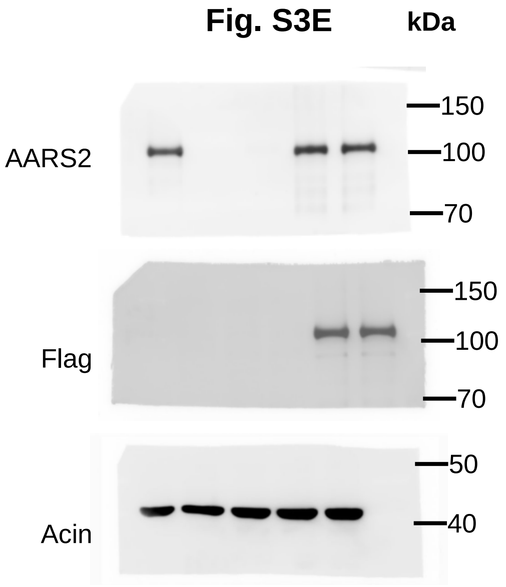

Source Data Figure S4A

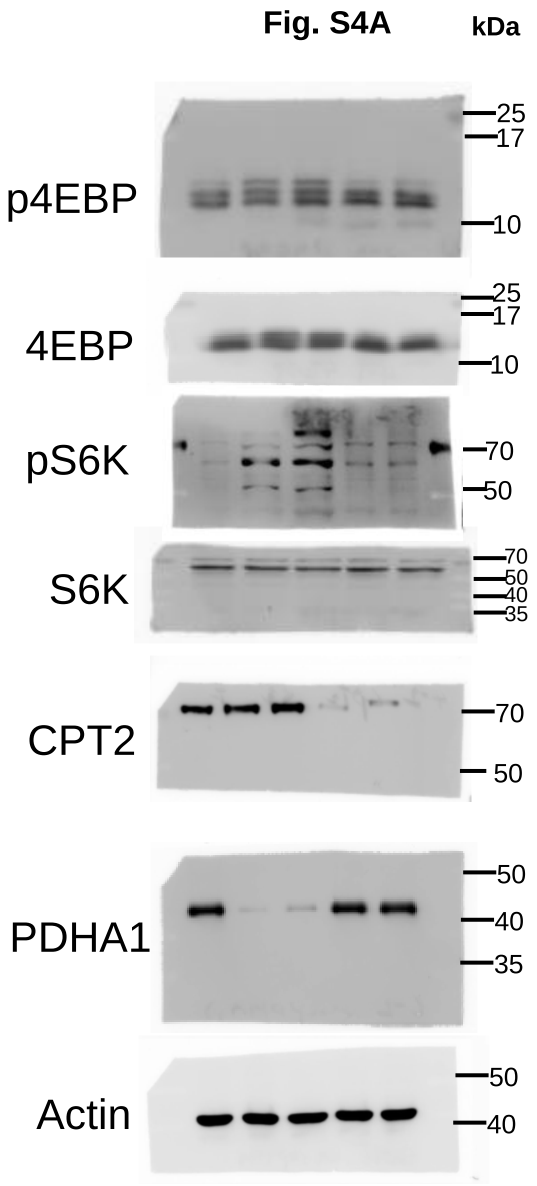

Source Data Figure S4B

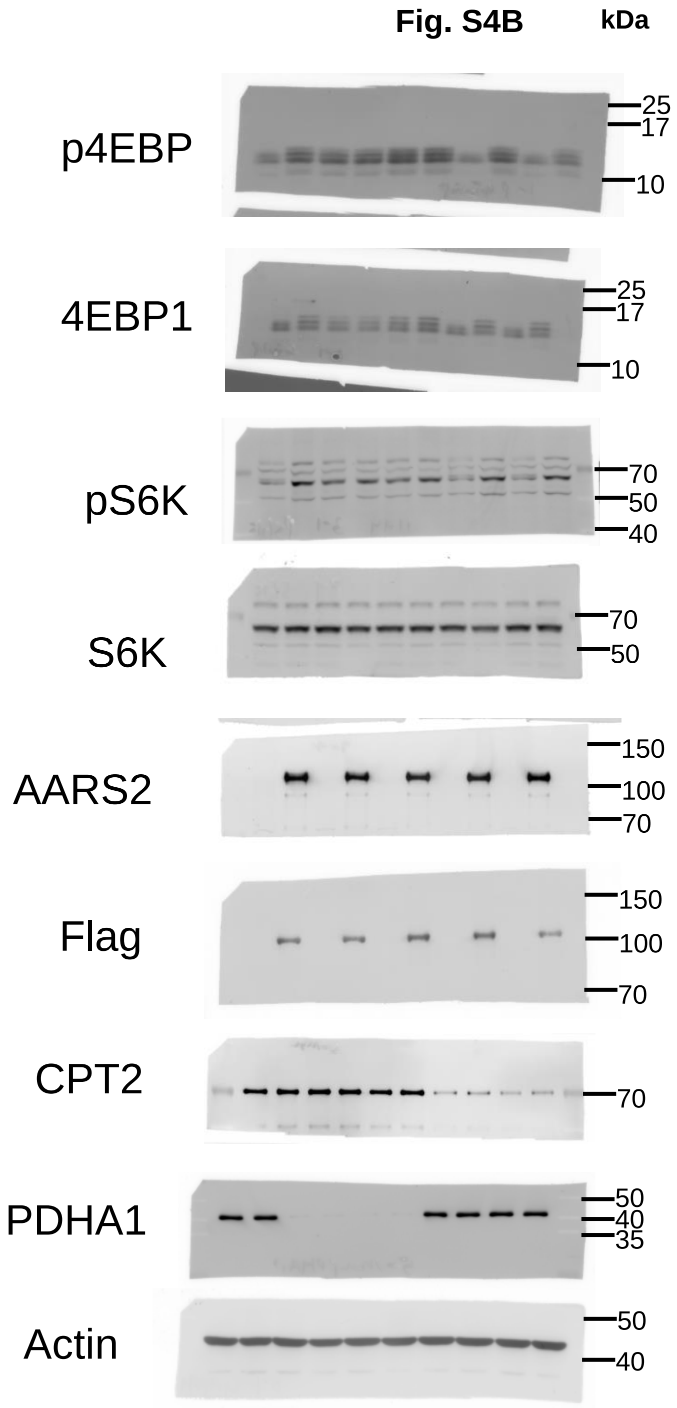

Source Data Figure S7A

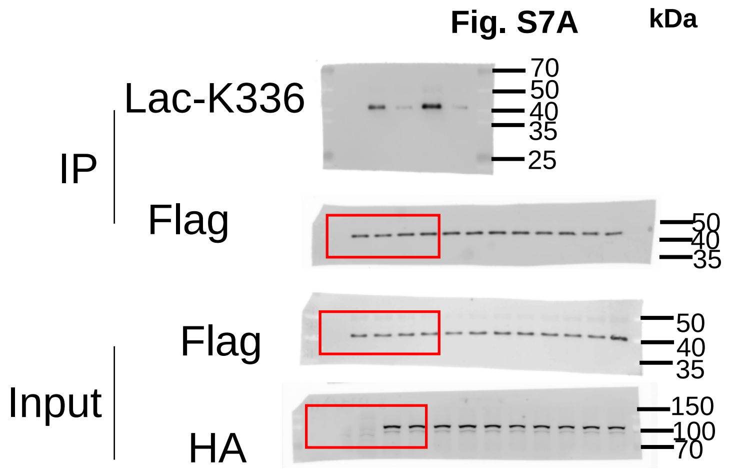

Source Data Figure S7B

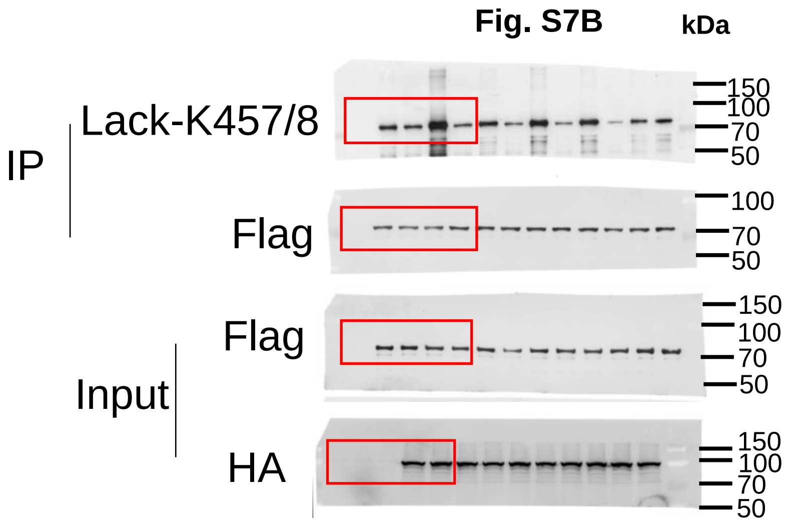

Source Data Figure S7D

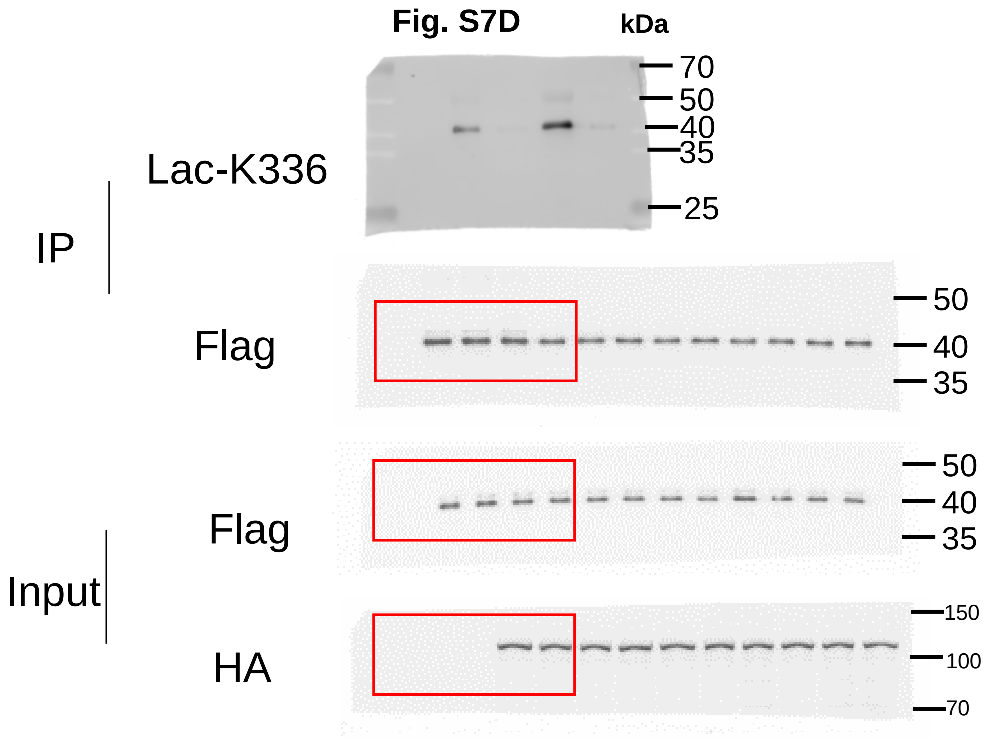

Source Data Figure S7E

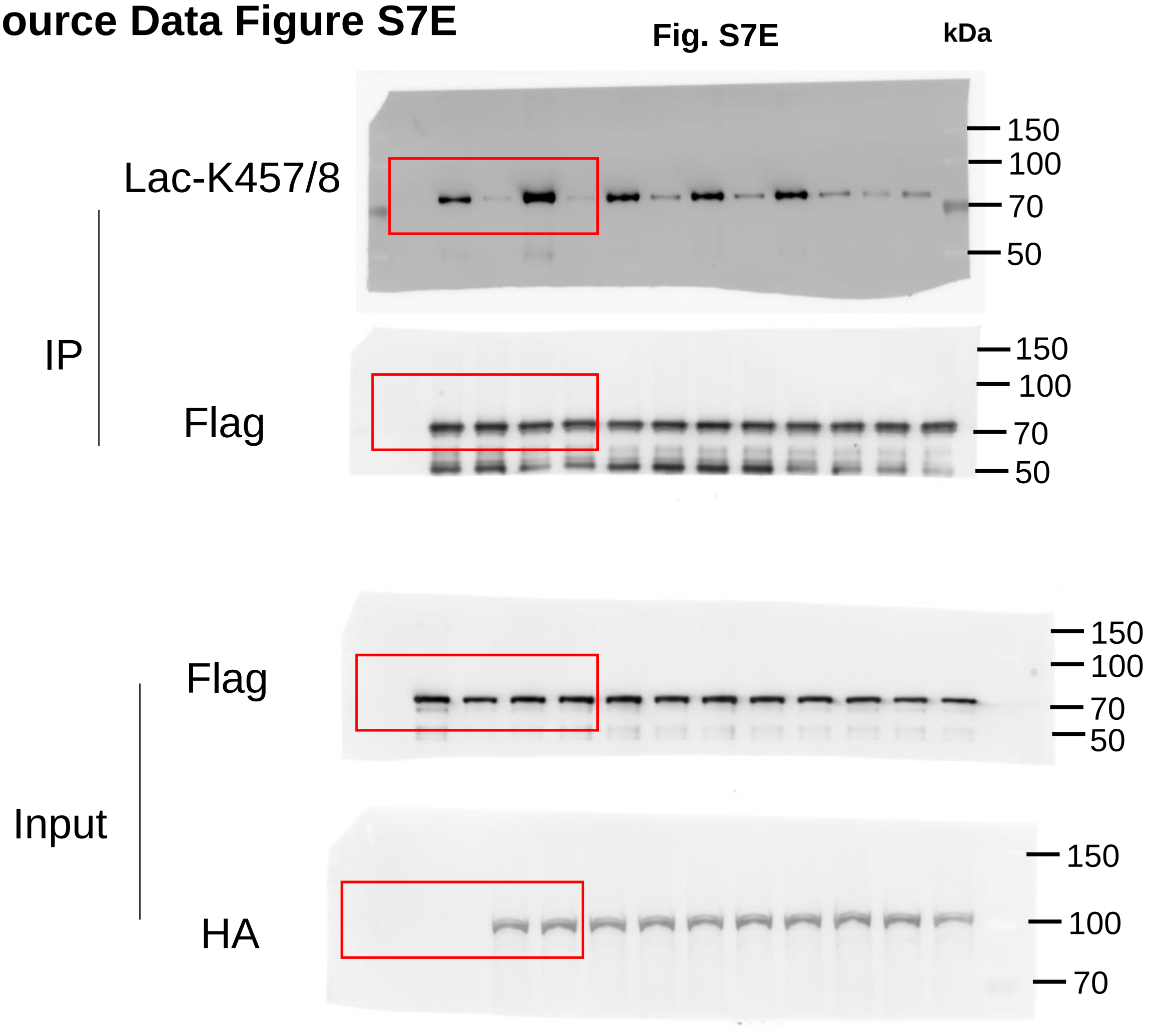

Source Data Figure S7F

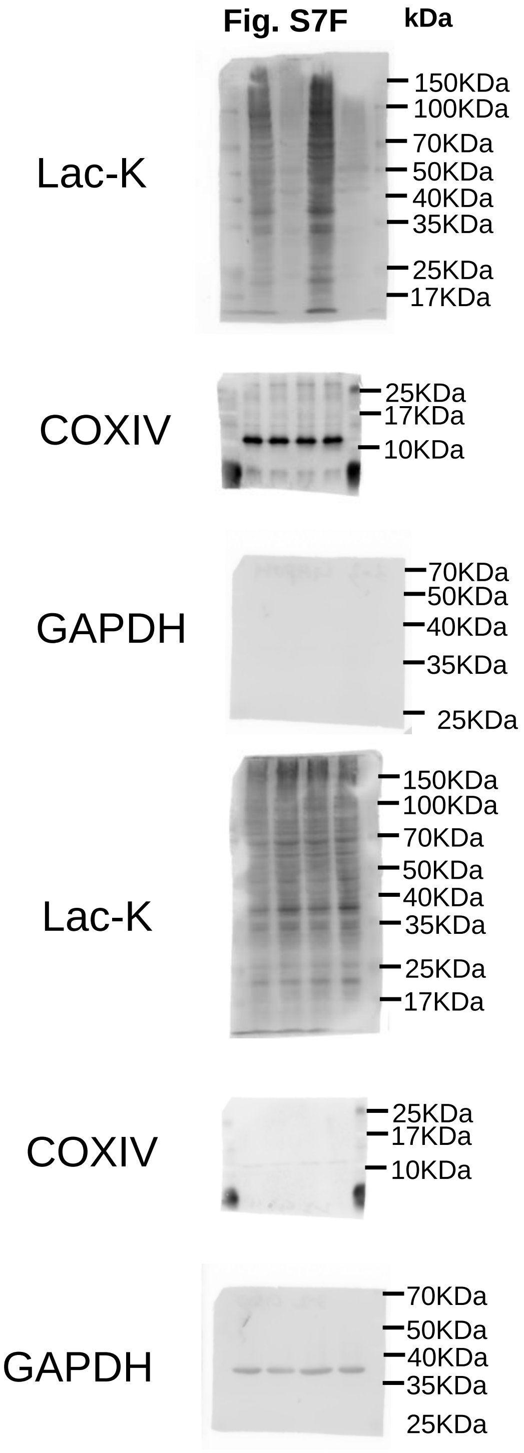

Source Data Figure S8A

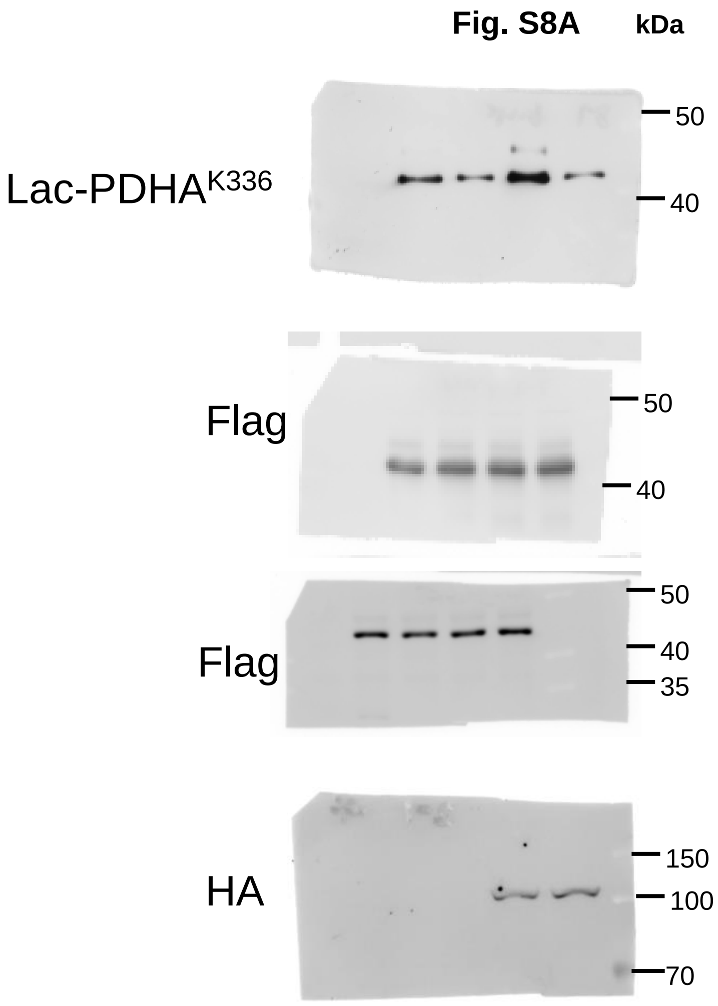

Source Data Figure S8B

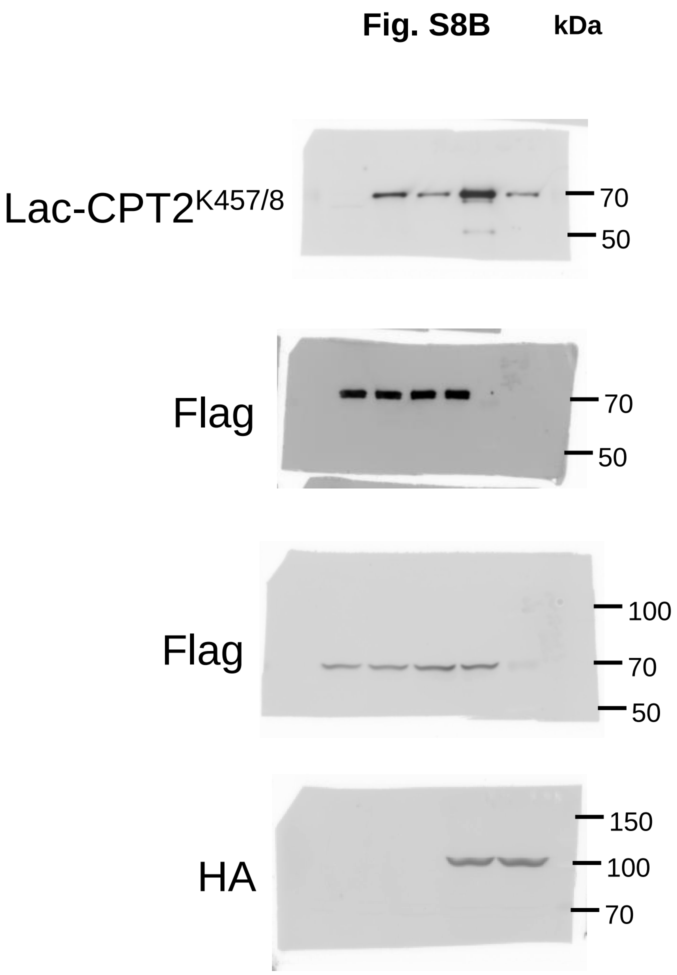

Supplement: Supplementary file 2 — Original Western blots [file 41420_2025_2501_MOESM2_ESM.pdf]
